# Electrostatic Self‐Assembly Induced Nest‐Like MXene Networks on Fabric for Ultra‐Broadband Flexible Absorption

**DOI:** 10.1002/advs.75438

**Published:** 2026-05-08

**Authors:** Min Luo, Zihao Chen, Haotian Li, Kexun Li, Wei Wang, Weishi Jiao, Donghong Wang, Qiye Wen

**Affiliations:** ^1^ School of Electronic Science and Engineering University of Electronic Science and Technology of China Chengdu China; ^2^ Tianfu Jiangxi Laboratory Chengdu China; ^3^ China‐Blarus Belt and Road Joint Laboratory on Electromagnetic Environment Effect The 33rd Research Institute of China Electronics Technology Group Corporation Taiyuan China; ^4^ Shenzhen Institute for Advanced Study University of Electronic Science and Technology of China Shenzhen China

**Keywords:** flexible fabric, MXene, superhydrophobicity, terahertz, ultra‐broadband absorption

## Abstract

Advancement of next‐generation information technologies is driving the increasing integration of millimeter‐wave and terahertz (THz) communication, detection, and artificial intelligence technologies, thereby creating a demand for multifunctional absorbing materials to address complex electromagnetic interference. In this study, a flexible WPU‐MXene@FC composite fabric with ultra‐broadband absorption, superhydrophobicity, and excellent durability is developed. The base fabric (FC) is modified via surface plasma treatment to introduce positively charged active sites on fibers. Driven by electrostatic interaction, negatively charged MXene is self‐assembled with modified FC. The electrostatic interactions process induces a “nest‐like” structure on fibers, building construct MXene multiple loss paths. Waterborne polyurethane (WPU) is finally coated to endow the FC with MXene oxidation protection, superhydrophobicity, and stability. Results show that a 1.8 mm‐thick WPU‐MXene@FC achieves an effective absorption bandwidth spanning 25.3–1200 GHz. Within 0.2–1.0 THz, the reflection loss (*RL*) value is below −30 dB, reaching a minimum of −45.2 dB. After 500 bending cycles, its *RL* remains below −30 dB. The WPU‐MXene@FC exhibits superhydrophobicity (contact angle 151.3°, sliding angle 1.2°), excellent air permeability, and flexibility. This multifunctional FC has important applications in wearable devices, communications, and provides strong support for the development of lightweight stealth structures and flexible electromagnetic camouflage.

## Graphical abstract

In this study, an electrostatically self‐assembled WPU‐MXene@FC flexible fabric constructs a “nest‐like” structure on fibers, building construct MXene multiple loss paths, achieving ultra‐wideband absorption of 25.3–1200 GHz in a thickness of 1.8 mm, while also exhibiting superhydrophobicity, acid and alkali resistance, and bending resistance.

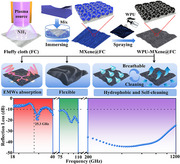

## 1 Introduction

With the rapid development of new‐generation information technologies, electronic devices are evolving toward higher frequencies and greater integration [1, 2, 3]. In particular, in cutting‐edge applications such as wireless communications, radar detection, and radio astronomy, operating frequencies have rapidly expanded from the microwave to the millimeter‐wave and terahertz (THz) bands [4, 5, 6]. However, electronic devices inevitably generate complex electromagnetic interference during operation, which not only degrades the performance of peripheral circuits and precision systems but may even cause harm to the human body [7, 8]. Electromagnetic absorbing materials (EAMs) effectively convert incident electromagnetic waves (EMWs) into thermal energy through mechanisms such as dielectric loss, magnetic loss, and interface polarization, fundamentally eliminating interference signals [9, 10]. Therefore, they are considered a key approach to achieving efficient electromagnetic compatibility [11, 12]. However, the absorption bandwidth of most materials is limited, making it difficult to cover the complex electromagnetic environment of the GHz–THz frequency band [13]. Second, current EAMs predominantly exist in the form of bulky foams or blocks, rendering them cumbersome for application in the packaging processes or conformal integration of electronic devices [14, 15]. Furthermore, EAMs integrated into electronic devices are typically attached to the exterior of the devices or systems and are frequently used in outdoor scenarios, which require a certain level of resistance to extreme environments, such as water repellency, self‐cleaning ability, and environmental friendliness, among others [16]. Ideal EAMs must not only achieve efficient absorption within an ultra‐wide frequency band but also have environmental adaptability, flexibility, and tailorability to meet the application needs of emerging fields such as wearable electronics, flexible devices, and intelligent protection systems. Nevertheless, simultaneously achieving ultra‐broadband (GHz–THz) absorption, conformal flexibility, and long‐term environmental robustness remains challenging.

MXene is a 2D layered material composed of transition metals (M) and carbides, nitrides, or carbonitrides (X), usually obtained by selective chemical etching and other methods to peel off its precursor MAX ceramics (A is the main group element, such as Al, Si) [17, 18]. Its high specific surface area and abundant polar functional groups can form a large number of dipole loss centers [19]. Our previous studies have shown that ultrathin MXene nanosheets with a thickness of 10–30 nm can achieve an intrinsic absorption limit of up to 50% in the 0.5–10 THz frequency band [20]. Based on this, MXene nanosheets can be widely used as EMWs absorbing agent and have been applied in various composite systems [21, 22, 23, 24, 25]. For example, Wan et al. [26] reported a MXene water‐based coating based on copolymer‐polyacrylic, which achieved a reflection loss (*RL*) of −32.8 dB on a foam sponge. Li et al. [27] prepared an ultra‐thin broadband MXene/rGO composite film with a minimum reflection loss (*RL_min_*) of −57.7 dB and an absorption bandwidth covering 0.37–2.0 THz. Although MXene has shown excellent performance in the field of terahertz absorption, its carbon‐based skeleton has the disadvantages of being brittle, easily oxidized, and having poor environmental tolerance, which limits its application in complex environments [28, 29]. In contrast, multifunctional fabric (FC) with the advantages of light weight, excellent absorption performance, good flexibility, ease of tailoring, and environmental friendliness, shows broad application prospects in daily life and specialized protection fields [30]. As the fundamental unit of textiles, fiber structures can be employed as skeletons for terahertz absorbing materials, not only achieving good impedance matching that allows more EMWs to effectively couple into and be dissipated within the material, but also providing a stable framework for the absorbers, thereby further enhancing the overall absorption performance [31, 32]. For example, Li et al. [33] used polydopamine and hexadecyltrimethoxysilane to modify the surface of carbon fiber FC, and its effective absorption bandwidth (*EAB*) can reach 6.2 GHz with a thickness of 2.2 mm. Xing et al. [34] proposed to construct a fabric composite material with SiC fiber as the electromagnetic loss phase, which exhibited an *EAB* of 8.78 GHz with a thickness of 2.8 mm. While the surface modification effectively enhances durability and impedance matching in a microwave window, the absorption remains concentrated in a limited frequency range. Furthermore, if FC can be endowed with multifunctional properties such as corrosion resistance and self‐cleaning while maintaining flexibility and high‐efficiency microwave absorption performance, its practical value and commercial potential in complex environments will be significantly enhanced [35, 36].

Here, we propose a simple and efficient strategy to induce positive charges on FC fibers through nitrogen plasma‐induced amine groups, thereby promoting MXene loading and preparing MXene@FC composite materials with high absorption efficiency. Meanwhile, the introduction of WPU coating into the FC creates multiple functionalities to the FC, including superhydrophobicity, self‐cleaning, and acid/alkali resistance. Specifically, nitrogen plasma activation introduces amine‐like surface functionalities that render the fiber surface cationic under the aqueous assembly conditions, enabling electrostatic adsorption self‐assembly of negatively charged MXene nanosheets and promoting the formation of a uniform, interconnected “nest‐like” structure at fiber junctions. The resulting WPU‐MXene@FC composite fabrics achieved near 100% absorption efficiency in the 0.2–1.2 THz (matching thickness 1.8 mm), with an *EAB* covering 25.3–1200 GHz and *RL_min_* of −45.2 dB. Importantly, the FC maintained excellent absorption performance and structural stability after acid/alkali immersion and multiple bending cycles. This study not only provides an effective strategy for the design of high‐performance terahertz absorbing materials but also introduces a novel concept of achieving multifunctional integration through MXene‐functionalized fabrics. Such flexible multifunctional composite fabrics hold broad application prospects in wearable smart electronics, hydrophobic sensors, and electromagnetic interference absorption.

## 2 Results and Discussion

**FIGURE 1:**
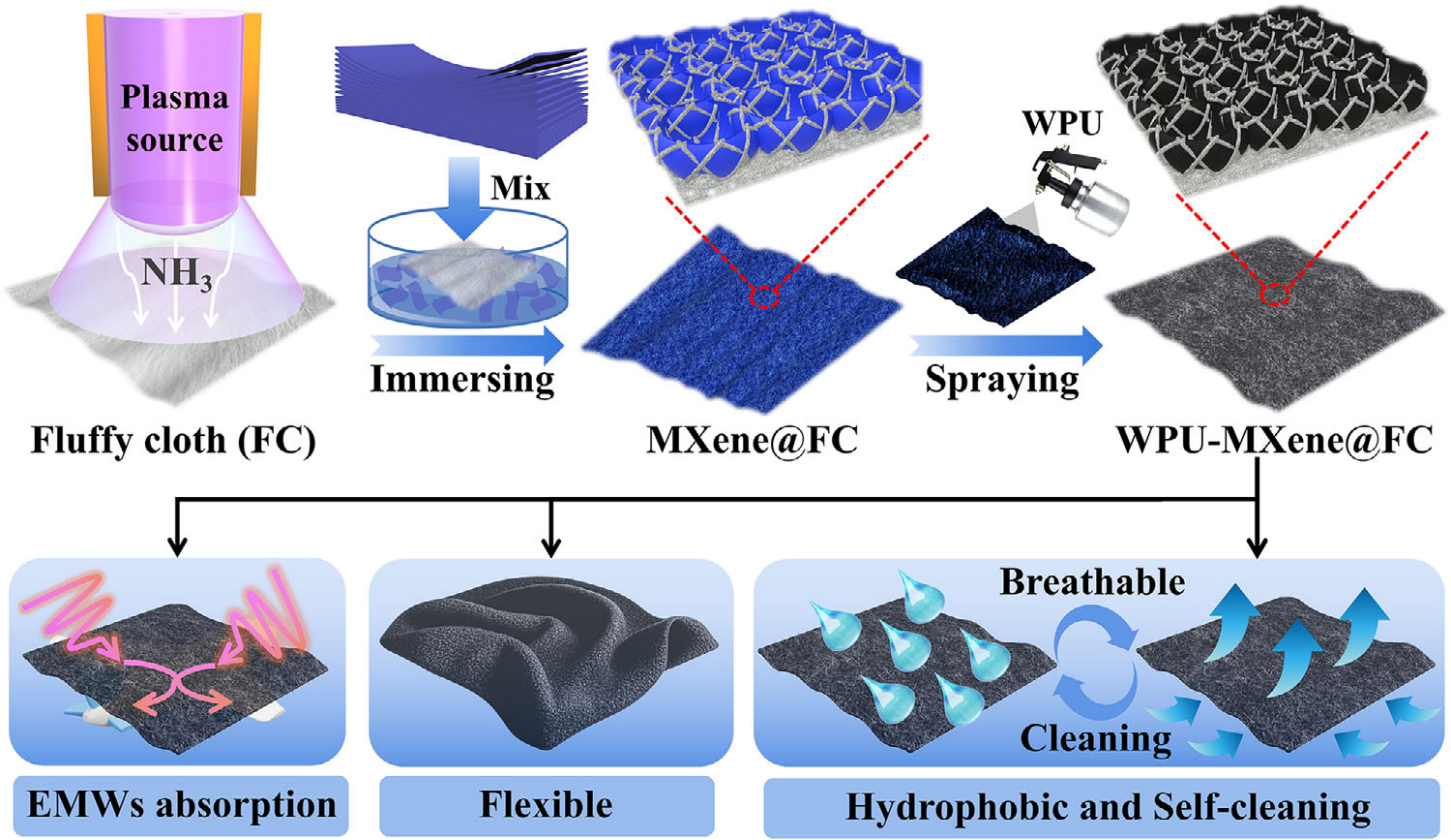
Preparation process and structural functional characteristics of WPU‐MXene@FC composite fabrics.

Figure 1 illustrates the preparation process and structural‐functional characteristics of the WPU‐MXene@FC composite fabrics. The fabrication protocol involves surface plasma treatment of the felt‐like FC substrate, synthesis of MXene dispersion, immersion of the treated substrate in the dispersion, and subsequent protection and hydrophobic modification via WPU emulsion spraying. Notably, the plasma‐treated felt‐based FC maintains a rich fibrous microstructure, as shown in Figure S1a,b. The chemical activation effect of this type of nitrogen plasma activation on the polyester surface has been confirmed by many studies, which can significantly improve the penetration and adhesion of subsequent functional layers [37, 38, 39]. The resulting surface charge facilitates the rapid adsorption of MXene nanosheets onto the fiber surfaces when immersed in MXene dispersion containing negatively charged functional groups (─OH, ─F, and ─Cl). Meanwhile, the synthesis of various MXene dispersions is mainly based on the fact that the M─X bond is a mixed covalent/metal/ionic bond, while the M─A bond is mainly a metallic bond, and the M─X bond strength is stronger than the others [40]. Therefore, different MAX require the use of appropriate etchants to selectively remove the “A” layer to obtain different types of multilayer MXene, and then prepare few‐layer MXene dispersions through intercalation, as shown in Figure S2. The preparation schemes of different MXene dispersions are summarized in Table S1. The prepared MXene dispersion samples are shown in Figure S3. It can be observed that MXene dispersions exhibit various optical properties, and the differences in elemental composition often result in distinct surface optical colors that can be visually identified in the dispersions. Moreover, when irradiated with a laser, the different MXene dispersions exhibited colloidal characteristics, as evidenced by the appearance of the Tyndall effect [41, 42].

Furthermore, during the adsorption of MXene, electrostatic interactions between the modified fibers led to the formation of numerous “nest‐like” structures, which remain intact during the WPU spray‐coating process and even become more stable due to the presence of WPU. The WPU emulsion employed in this work exhibits low surface energy, excellent corrosion resistance, and strong water repellency properties. As the WPU layer was coated on the surface of FC fibers, it not only enhanced the interfacial interaction between MXene nanosheets and the FC fibers but also protected the MXene from detachment, oxidation, and degradation, thereby improving the overall resistance to chemical corrosion and mechanical fatigue [43, 44]. As we will discuss below, the WPU‐MXene@FC composite fabrics prepared in this study not only achieved efficient and broadband absorption of EMWs but also exhibited flexibility, breathability, and self‐cleaning properties.

### 2.1 Phase and Microstructures

**FIGURE 2:**
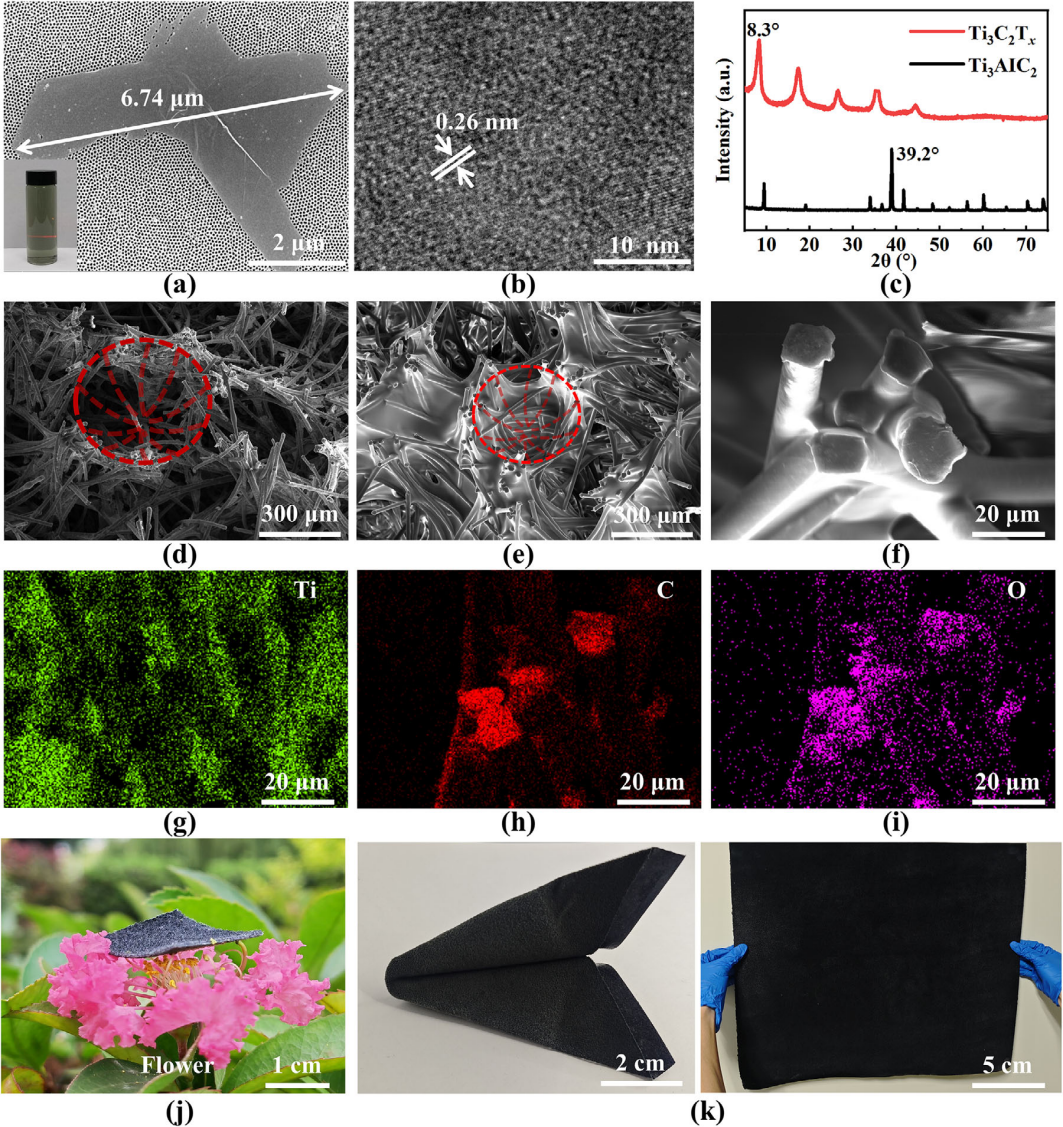
(a) SEM image of MXene nanosheets. (b) HRTEM image of MXene dispersion. (c) XRD characterization of MAX phase and MXene. (d) Surface SEM image of MXene@FC. (e) Surface SEM image of WPU‐MXene@FC. (f) SEM characterization of multiple WPU‐MXene@FC fibers. (g–i) EDS mapping of Ti, C, and O elements of multiple WPU‐MXene@FC fibers, respectively. (j) Optical image of WPU‐MXene@FC placed on a pistil. (k) Folding characteristics and large‐area optical image of WPU‐MXene@FC.

Figure 2a shows the SEM image of the prepared few‐layer MXene nanosheet, and the lateral size of the prepared MXene nanosheet is 6.74 µm. In addition, Figure 2b shows the high‐resolution transmission electron microscopy (HRTEM) image of the MXene nanosheet. The HRTEM results show that the MXene nanosheet is very thin and transparent, with a lattice constant of approximately 0.26 nm [45]. Transmission electron microscopy (TEM) tests of the MXene nanosheet further confirmed the MXene nanosheet and its single crystal structure with a hexagonal base lattice (Figure S4). Figure 2c shows the XRD patterns of Ti_3_AlC_2_ (MAX) and Ti_3_C_2_T_x_ (MXene). It can be seen that after etching, the characteristic peak (39.2°) of the original Ti_3_AlC_2_ disappears, and the characteristic peak (8.3°) of Ti_3_C_2_T_x_ appears, which fully demonstrates that MAX has been converted into MXene [46], further proving that the Al phase has been etched away. These results indicate that a high‐quality MXene dispersion has been successfully prepared [47].

Figure S5 compares the surface morphologies of MXene@FC and WPU‐MXene@FC. The WPU‐treated FC exhibits a smoother surface, while the surface morphology remains unchanged. In addition, Figure 2d shows the surface SEM image of the MXene@FC, where MXene nanosheets were firmly adsorbed on the fiber surface of the FC under electrostatic attraction, bridging adjacent fibers and forming “nest‐like” structure. These “nest‐like” structures play a pivotal role in promoting the EMWs absorption—they enable the waves penetrating into the fibers to undergo multiple reflections and scattering, and further facilitate their progressive absorption or attenuation by the MXene nanosheets during transmission. Moreover, abundant MXene film structures were formed within the FC, thereby enhancing the absorption loss characteristics of the material [48]. The streaming zeta‐potential measurements further confirm the electrostatic self‐assembly properties of the modified FC (Figure S6). At pH = 6, the modified FC exhibits a positive zeta potential (+14.24 mV), while MXene@FC carries a negative charge (−10.07 mV), consistent with the electrostatic adsorption of negatively charged MXene onto the plasma‐activated fibers. Figure 2e shows an SEM image of the MXene@FC surface after WPU treatment. From a detailed perspective, the MXene film is covered by a thin layer of WPU polymer, resulting in reduced fiber surface roughness. The SEM structure of the WPU‐MXene@FC composite fabrics is the same as that of the MXene@FC, indicating that the addition of the WPU solution does not affect the dispersion structure of the MXene nanosheets in the flannel. In addition, Figure 2f shows an SEM image of multiple fiber hairs of the WPU‐MXene@FC composite fabrics. It can be seen that the WPU layer adheres to the fiber surface and enhances the interaction between the MXene nanosheets and the fibers. To further demonstrate the internal structural characteristics of WPU‐MXene@FC, EDS spectra of Ti, C, and O elements were analyzed in WPU‐MXene@FC, as shown in Figure 2g–i. Due to the strong intermolecular forces between MXene and the polymer fibers, the fibers can be uniformly coated with the fibers. Ti is uniformly distributed on the surface of the fibers, indicating that the fibers are coated with MXene nanosheets [49]. Figure S7 shows a high‐resolution surface SEM of WPU‐MXene@FC, which shows that MXene nanosheets form a relatively continuous sheet coating on the fiber surface and construct bridging and connecting structures at fiber intersections. The 2 × 2 cm^2^ WPU‐MXene@FC in Figure 2j can be easily placed on the pistil without bending it, fully demonstrating its extremely low areal density and excellent lightweight properties. Furthermore, the prepared WPU‐MXene@FC composite fabrics are foldable and capable of large‐scale fabrication, as shown in Figure 2k. This demonstrates the high flexibility of the FC, allowing it to be rolled up or even folded without any damage, while also possessing a large specific surface area.

### 2.2 Broadband Electromagnetic Absorption Properties of WPU‐MXene@FC

The electromagnetic absorption properties of composite fabrics were systematically investigated, and their absorption mechanism was revealed. The FC used is a naturally wave‐transmitting material. The original FC transmits incident terahertz waves almost completely, with virtually no reflection (Figure S8). Compared to cotton FC, the fiber fleece exhibits higher transmittance, indicating superior impedance matching. However, after both types of fabrics were impregnated with MXene dispersion, their transmittance to THz dropped significantly, approaching zero. Notably, the reflectivity of cotton fabrics increased significantly during this process, potentially leading to secondary contamination from reflected EMWs. Further research revealed that the absorption efficiency of MXene@ML decreased significantly at low frequencies. This is due to the low fiber density within cotton fabrics, which results in an impedance mismatch with free space at low frequencies, generating significant electromagnetic reflections. In contrast, MXene@FC exhibits excellent electromagnetic absorption performance, with an absorption efficiency close to 100%. This result indicates that using the fiber microstructure as the skeleton of MXene can achieve good impedance matching, allowing more incident EMWs to be effectively coupled into the material and absorbed [50]. The main mechanism is that a continuous and rich MXene film is formed on the surface of the fiber, causing the incident EMWs to undergo a multiple attenuation process of “absorption‐reflection‐reabsorption” in the FC, thereby significantly enhancing the overall absorption effect [51].

**FIGURE 3:**
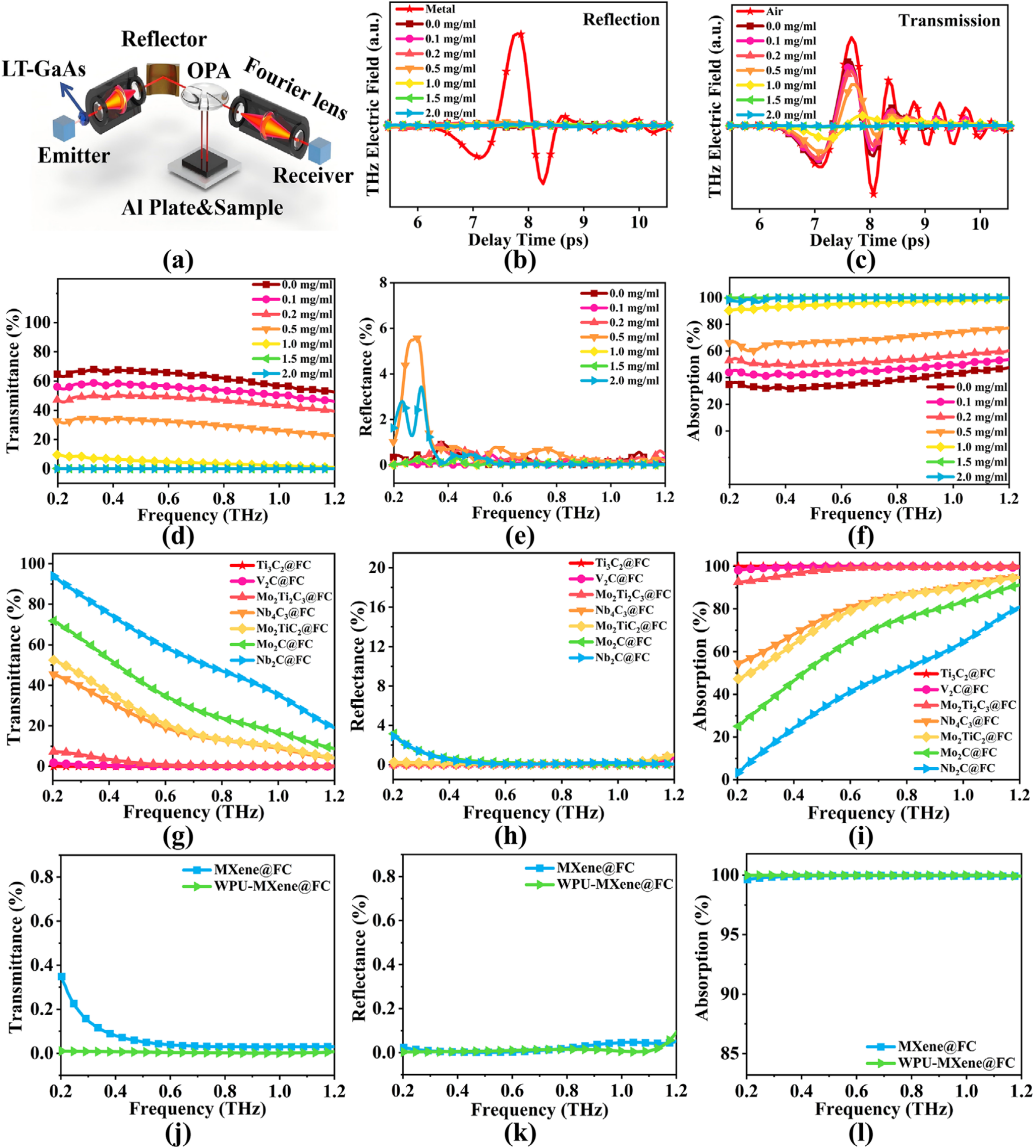
(a) Schematic diagram of THz‐TDS measurement. (b, c) THz reflection and transmission time‐domain spectra of MXene@FC composite fabrics at different MXene concentrations, respectively. (d–f) Transmission, reflection, and absorption efficiencies of MXene@FC composite fabrics at different MXene concentrations at 0.2–1.2 THz, respectively. (g–i) Transmission, reflection, and absorption efficiencies of MXene@FC composite fabrics at 0.2–1.2 THz, respectively, at different MXene types. (j–l) Transmission, reflection, and absorption efficiencies of MXene@FC and WPU‐MXene@FC composite fabrics at 0.2–1.2 THz, respectively.

Figure 3a illustrates the working principle of the terahertz time‐domain spectroscopy (THz‐TDS) system. The femtosecond excitation laser illuminates a low‐temperature‐grown GaAs (LT‐GaAs) photoconductive antenna (PCA) to generate a broadband THz pulse. The THz beam is then guided and focused onto the sample using the Fourier prism and off‐axis parabolic mirrors (OPA). The transmitted and reflected THz signals are coherently detected and recorded as time‐domain waveforms [52]. Based on this test platform, the effect of the MXene dispersion concentration on the electromagnetic absorption properties of the WPU‐MXene@FC composite fabrics was studied, as shown in Figure 3b,c. As the MXene concentration gradually increases, the field intensity in the terahertz transmission time domain spectrum is significantly weakened, while the reflection spectrum intensity almost decays to zero. Furthermore, the reflectance, transmittance, and absorptivity of samples with different dispersion concentrations were calculated in the frequency range of 0.2–1.2 THz (Figure 3d–f). As the concentration of the MXene dispersion increased from 0 to 2.0 mg/mL, the transmittance decreased significantly, while the reflectance remained at a low level across most frequency bands. Simultaneously, the absorption efficiency monotonically increased, reaching 99.9% at concentrations ≥1.5 mg/mL. This is because higher loadings enhance ohmic losses and interfacial polarization within the interconnected MXene network, thereby suppressing transmission and enhancing overall terahertz absorption. Furthermore, the effect of MXene type on the electromagnetic absorption properties of the composite fabrics was investigated. At a fixed concentration of 1.5 mg/mL, the effects of seven MXenes —Ti_3_C_2_, V_2_C, Mo_2_TiC_2_, Nb_4_C_3_, Mo_2_Ti_2_C_3_, Mo_2_C, and Nb_2_C on the transmission, reflection, and absorption efficiencies of the composite fabrics were tested (Figure 3g–i). It is clearly evident that Nb_2_C@FC exhibits strong transmission, reaching a high efficiency of 92.4% at 0.2 THz. In contrast, Ti_3_C_2_@FC and V_2_C@FC exhibited transmission efficiencies below 0.1% across the entire tested frequency band, demonstrating excellent EMWs blocking capabilities. Meanwhile, Nb_2_C@FC exhibits relatively high reflectivity, but its reflection efficiency remains below 5%. Overall, Ti_3_C_2_@FC exhibits the best absorption characteristics across the entire frequency band, with an overall absorption efficiency exceeding 99.9%. These results demonstrate that the electromagnetic response of the FC can be effectively tuned by manipulating the MXene dispersion concentration and type. Based on the above research results, Ti_3_C_2_ is preferred as a representative MXene.

This work compares the electromagnetic transmission, reflection, and absorption properties of MXene@FC and WPU‐MXene@FC, as shown in Figure 3j–l. Clearly, the introduction of the WPU solution did not reduce the absorption loss capacity of the MXene@FC fabric. On the contrary, the addition of WPU solution reduced the transmission efficiency of the low‐frequency part and increased the absorption loss capacity. This is because the WPU layer on the surface can improve the impedance matching of the MXene@FC, allowing low‐frequency EMWs to enter the flannel [53]. The WPU layer on the surface also enhances the interaction between MXene and fibers, generating more interfacial polarization, which increases the conductive loss caused by MXene [54]. Therefore, the reflection efficiency of the tested WPU‐MXene@FC composite fabrics is lower. To further elucidate the absorption mechanism of the composite fabric, we analyzed and tested the complex dielectric parameters of WPU‐MXene@FC in the 200–1200 GHz range and the resulting impedance matching and attenuation capabilities (Figure S9). Since the material system contains no magnetic components, µ_r_≈1 can be approximated, and absorption is mainly dominated by dielectric loss. The corresponding dielectric loss tangent tanδ_ε_ = ε″/εʹ is approximately 0.28–0.38, indicating that the material possesses a stable electrical dissipation channel over a wide frequency range. Further calculations under the condition of µ_r_≈1 yielded a normalized intrinsic impedance |Z/Z_0_| of approximately 0.74–0.88, indicating that the material has good coupling capability for incident EMWs over a wide frequency range [55]. Simultaneously, the attenuation constant α increases significantly with frequency, indicating that EMWs can be more effectively attenuated and dissipated after entering the material.

**FIGURE 4:**
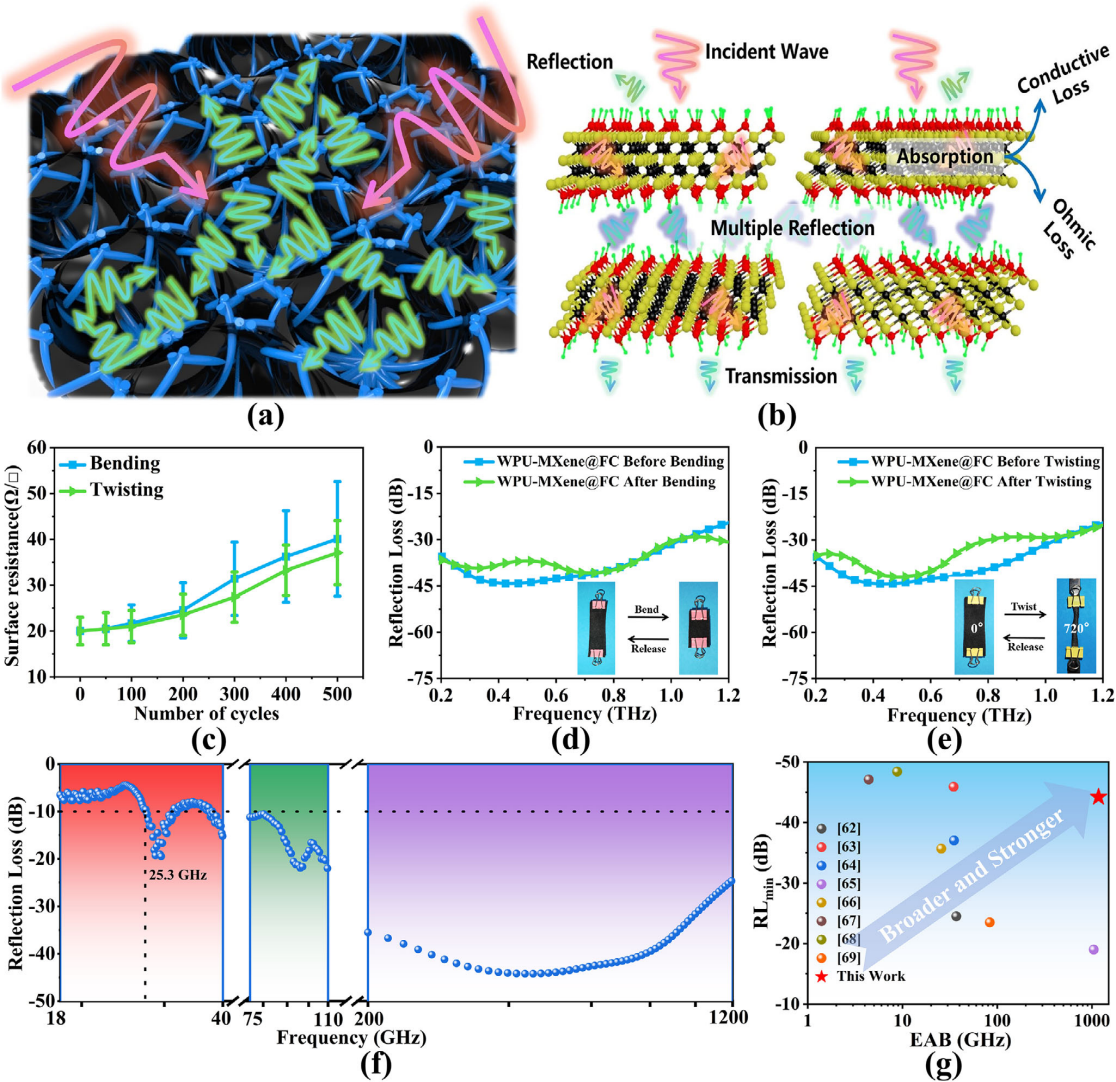
(a, b) Electromagnetic absorption mechanism of the synergistic effect of the fiber microstructure and MXene nanosheets of WPU‐MXene@FC. (c) Relationship between the surface resistance of WPU‐MXene@FC composite fabrics and 500 bending and twisting cycles. (d) *RL* of WPU‐MXene@FC before and after 500 bending cycles. (e) *RL* of WPU‐MXene@FC before and after 500 twisting cycles. (f) *RL* curve of WPU‐MXene@FC in the range of 18–1200 GHz. (g) Comparison of the *EAB* and *RL_min_* of WPU‐MXene@FC with other absorption materials [13, 32, 62, 63, 64, 65, 66, 67].

Based on the above results, the electromagnetic absorption mechanism of the WPU‐MXene@FC composite fabrics can be inferred, as illustrated in Figure 4a,b. The WPU‐MXene@FC composite fabrics exhibited excellent EMWs absorption performance, primarily attributed to the synergistic effect between the fiber microstructure and MXene nanosheets. At the fiber microscale, the porous and 3D architecture of the fibers offers numerous anchoring sites for MXene deposition. The abundant interfaces formed between MXene and the fibers act as polarized capacitor‐like structures, dissipating incident EMWs through conduction loss. In addition, this “nest‐like” structure not only effectively improves impedance matching, allowing the incident EMWs to enter the material smoothly, but also increases the propagation path of the EMWs in the FC and the opportunity for multiple scattering, thereby significantly improving the attenuation efficiency. Second, considering the electrical characteristics of MXene nanosheets, their lateral structure enables carrier migration upon EMWs incidence, generating microcurrents and inducing ohmic losses that dissipate the incident electromagnetic energy [20, 26]. Moreover, under the action of the alternating electromagnetic field, a large amount of interfacial polarization is generated between the MXene nanosheets and the fiber matrix, and its intrinsic conductive loss further converts the electromagnetic energy into thermal energy, achieving multiple energy dissipation [56]. Moreover, the layered structure of MXene provides channels for electron transport and phonon scattering, which facilitates the formation of a continuous electromagnetic loss network [57]. Finally, from the perspective of macroscopic electromagnetic response, the EMWs within the composite fabrics undergo a multi‐stage attenuation process of “absorption‐reflection‐reabsorption”. The incident EMWs are first absorbed by the MXene film layers, while the residual reflected waves are scattered within the porous fiber structure, re‐coupled into the material, and further absorbed [58]. This multiple loss mechanism significantly reduces *RL* and ensures nearly 100% efficient absorption of the material within the 0.2–1.2 THz frequency range.

To further verify the effectiveness of electrostatic self‐assembly, we prepared two sets of MXene@FC control samples: one with self‐assembly and one without, and performed SEM characterization (Figure S10a,b). Obviously, the self‐assembled samples formed a more uniform and continuous coverage of MXene on the fiber surface and intersections, and constructed a “nest‐like” structure with obvious 3D connectivity. In contrast, the samples after soaking mainly show random accumulation and local reunion, uneven coverage, and weak connectivity. These morphological differences were further reflected in the THz absorption performance (Figure S10c). The MXene@FC with self‐assembly samples showed lower overall *RL* in the 0.2–0.95 THz range, while the soaked samples showed insufficient absorption in the low‐frequency range, which gradually increased with increasing frequency. In summary, electrostatic self‐assembly helps promote the uniform deposition of MXene in the fiber network and the formation of a 3D connected structure, thereby enhancing electromagnetic wave dissipation and improving overall absorption performance.

Considering that absorbing fabrics may encounter complex and variable environmental conditions in practical applications, the mechanical properties of the WPU‐MXene@FC composite fabrics were investigated to ensure sufficient toughness and durability for different usage scenarios. Figure S11 shows its torsion and bending resistance. The results show that even after a 720° torsion, the FC maintains its overall morphology intact and exhibits no structural damage, demonstrating excellent flexibility. To further evaluate its ultimate mechanical properties, uniaxial tensile tests were performed on WPU‐MXene@FC (Figure S12). The sample was cut into a dumbbell shape to ensure that the fracture occurred in the middle area, thereby avoiding stress concentration at the clamping end [59]. The test results showed that as the applied load increased, the FC gradually deformed until it broke when the tension increased to 36 N, with a fracture strain of up to 140%. This excellent ductility is attributed to the fact that the fibers are composed of polymer chains, and the chains form a stable network through chemical bonds and physical forces. Under the action of external forces, they can undergo significant stretching and deformation without breaking easily [60]. At the same time, the WPU's interwoven structure between the fibers further enhances the overall strength and toughness of the material. Furthermore, to investigate the stability of its electromagnetic absorption performance under cyclic loading, the surface resistance of the composite fabrics under repeated bending and torsion was further characterized (Figure 4c). The results showed that after 200 cycles, the surface resistance only increased slightly. After 300 cycles, the surface resistance slowly increased from an initial 20.2±4.2 Ω/□ to 37.1±6.4 and 41.2±8.2 Ω/□. This stability is primarily attributed to the strong interfacial interaction between the fleece fibers, MXene nanosheets, and the WPU coating. The WPU layer not only provides flexible protection but also effectively mitigates the risk of delamination or fracture between the fibers and the MXene. Considering the sensitivity of conductive properties to electromagnetic absorption attenuation [61], the *RL* changes of the composite fabrics before and after 500 bends and twists were further compared (Figure 4d,e). The results show that there is almost no significant difference in *RL* before and after the cycle, indicating that the change in conductivity is limited. This shows that the MXene nanosheets inside the composite fabrics are still firmly attached to the fiber surface and maintain an effective EMWs dissipation function, thus giving the FC excellent anti‐bending and stable absorption properties. The effect of the number of fabric stacking layers (1–3 layers) on the absorption *RL* in the THz band was further investigated, and the results are shown in Figure S13. Overall, the single‐layer sample can maintain a low *RL* in the range of 0.2–1.2 THz, indicating that the incident EMWs is sufficiently attenuated in the single‐layer structure. Increasing the number of stacking layers changes the equivalent thickness of the absorber, thereby modulating the EMWs impedance matching and phase conditions, introducing more matching modes and multiple reflection paths, which is manifested in the appearance of more peak frequencies in the *RL* curve and enhanced absorption in some frequency bands.

Furthermore, the electromagnetic *RL* of the prepared 1.8 mm thick WPU‐MXene@FC composite fabrics was tested in the 18–40, 75–110, and 200–1200 GHz (Figure 4f). Notably, the WPU‐MXene@FC exhibits ultra‐wideband absorption loss capability with a reflection loss generally below −10 dB above 25.3 GHz. In particular, the *RL_min_* measured at 439.6 GHz is −44.2 dB, at which point EMWs are more easily coupled into the fabric interior and attenuated in the MXene conductive network through ohmic loss and interfacial polarization dissipation. As the frequency increases from the W‐band to above 200 GHz, the current response of the MXene conductive network is enhanced at higher frequencies, and the proportion of conductive loss increases. At the same time, the shortening of the wavelength enhances multiple scattering and reflection within the porous fiber skeleton, increasing the propagation path and residence time, thereby further reducing reflection loss and improving absorption performance. As shown in Figure 4g and Table S1, compared with other reported absorbers [13, 32, 62, 63, 64, 65, 66, 67], the WPU‐MXene@FC composite fabrics achieve ideal ultra‐broadband low‐reflection absorption performance. Its absorption bandwidth, minimum reflection loss, and thickness performance are superior to most reported absorbers, while maintaining its softness, providing a new solution for high‐frequency and cross‐band electromagnetic protection.

### 2.3 Superhydrophobic and Acid and Alkali Resistance of WPU‐MXene@FC

**FIGURE 5:**
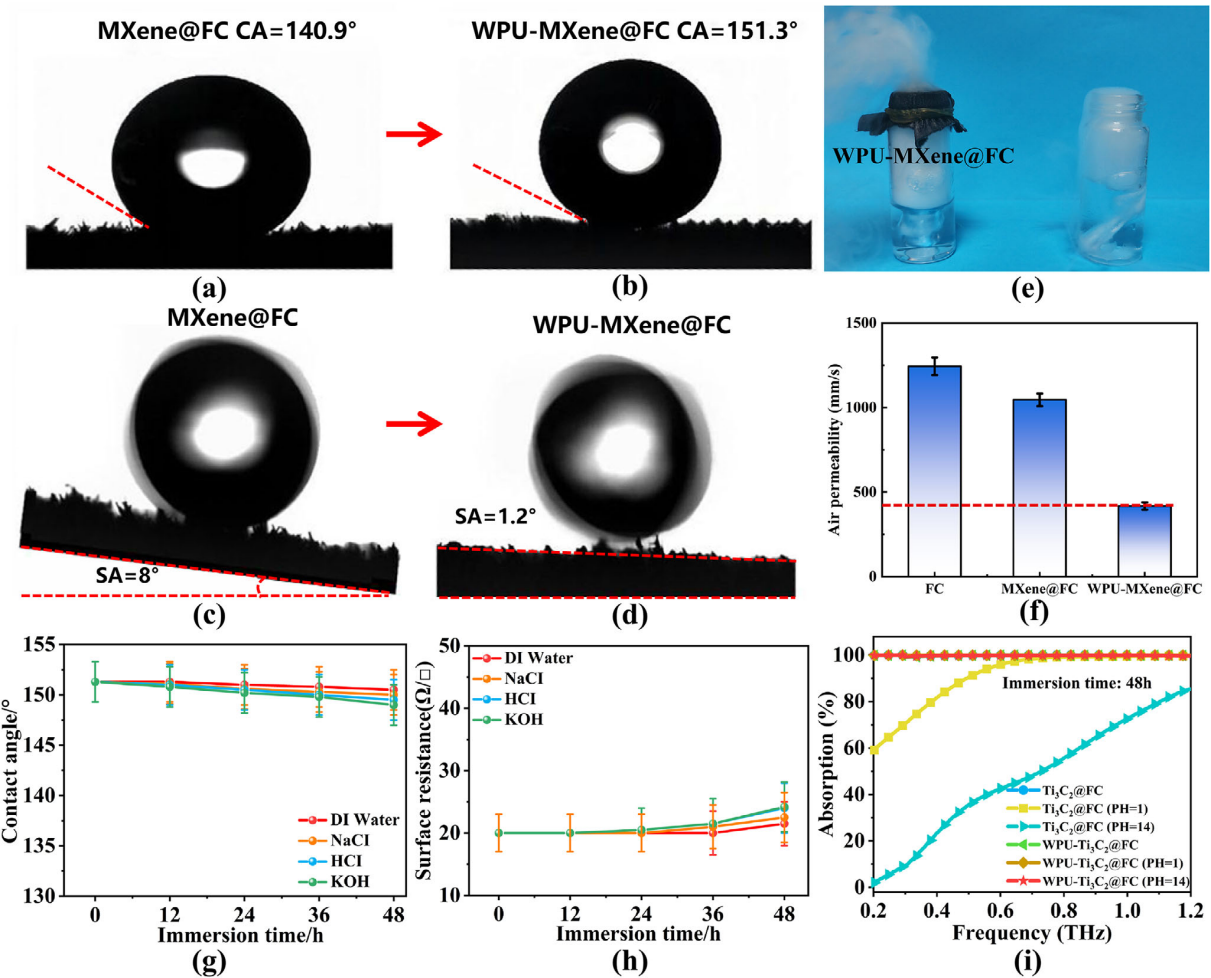
(a) CA of MXene@FC. (b) CA of WPU‐MXene@FC. (c) SA of MXene@FC. (d) SA of WPU‐MXene@FC. (e) Demonstration of the breathability of WPU‐MXene@FC. (f) Air permeability of three fabrics. (g) CA of WPU‐MXene@FC during immersion in DI water, sodium chloride, hydrochloric acid, and potassium hydroxide solutions. (h) Surface resistance of WPU‐MXene@FC after immersion in DI water, sodium chloride, hydrochloric acid, and potassium hydroxide solutions. (i) Terahertz absorption performance of MXene@FC and WPU‐MXene@FC after immersion in hydrochloric acid and potassium hydroxide solutions for 48 h.

FCs with hydrophobic properties are more suitable for outdoor humid environments, marine salt spray protection, and other application scenarios, and can still maintain a stable electromagnetic protection effect under complex working conditions. Here, the Laplace‐Young method [68] was used to test the water contact angle (CA) of MXene@FC and WPU‐MXene@FC composite fabrics, as shown in Figure 5a,b. Obviously, the CA of the MXene@FC composite fabrics after WPU treatment increased from the original 140.9° to 151.3°, realizing an absorbing composite fabric with superhydrophobicity. The increase in the CA is consistent with the conjecture of the design scheme. The MXene surface has rich hydrophilic functional groups, and the WPU solution has superhydrophobicity and low surface energy. By spraying the WPU solution, a hydrophobic protective layer is formed on the surface of the composite fabrics. Most of the MXene nanosheets and WPU are adsorbed on the surface of the fiber, which can effectively increase the superhydrophobicity of the composite fabrics. Next, the rolling angles of the MXene@FC and WPU‐MXene@FC composite fabrics were measured to evaluate their self‐cleaning properties, as shown in Figure 5c,d. The sliding angle (SA) is the critical angle between the inclined surface and the horizontal plane at which a droplet rolls on the inclined surface. The measured SA for the MXene@FC composite fabrics is 8°, while that for the WPU‐MXene@FC composite fabrics is 1.2°. The composite fabric's surface coated with the WPU solution exhibits extremely strong hydrophobicity. In Video S1, the liquid dropped onto the WPU‐MXene@FC composite fabrics can quickly slide off the surface of the composite fabrics, showing obvious waterproofness. In this process, impurities on the surface of the composite fabrics can also be removed, satisfying the self‐cleaning properties of the FC. Furthermore, Figure 5e,f experimentally studies the air permeability of WPU‐MXene@FC composite fabrics. A glass bottle filled with dry ice and DI water releases a large amount of white mist due to the rapid evaporation of dry ice. The amount of mist permeation was observed by wrapping the glass bottle with FC, and a quantitative test was performed. The results show that the air permeability of WPU‐MXene@FC reached 417.4 ± 22 mm/s, which is lower than that of untreated FC, but still higher than the air permeability index of breathable clothing (180 mm/s) [69].

In practical applications, the chemical stability of the material surface is also crucial [70]. To this end, this study simulated the harsh environment by immersing the WPU‐MXene@FC composite fabrics in DI water, sodium chloride solution, hydrochloric acid, and potassium hydroxide solutions to evaluate its corrosion resistance (Figure 5g). The results show that the CA of WPU‐MXene@FC gradually decreases with the extension of immersion time, but remains at a high level overall. For example, in potassium hydroxide solution, the CA decreases fastest, decreasing from 151.3° to 147.8° after 48 h of treatment. This slow decay is mainly due to the gradual desorption or hydrolysis of WPU molecules in acidic and alkaline environments, resulting in a weakening of the protective effect. At the same time, the surface resistance change of the sample during the immersion process was tested (Figure 5h). The initial surface resistance was 20.2 ± 4.2 Ω/□, and after 48 h of treatment, it increased to 21.5 ± 4.5 Ω/□ (DI water), 22.7 ± 5.2 Ω/□ (sodium chloride), 24.1 ± 5.5 Ω/□ (hydrochloric acid), and 24.3 ± 5.6 Ω/□ (potassium hydroxide), respectively. Within the first 24 h, the resistance change was relatively stable, but it increased significantly in the subsequent stage, further confirming the phenomenon of gradual hydrolysis of WPU in an acidic and alkaline environment. The increase in surface resistance will lead to a decrease in conductivity, which may weaken the absorption and attenuation ability of EMWs. To verify the retention of its absorption properties, the terahertz absorption performance of MXene@FC and WPU‐MXene@FC after immersion in strong acid and strong base solutions for 48 h was compared (Figure 5i). The results show that the absorption capacity of MXene@FC decreases significantly after treatment, especially under alkaline conditions, where the MXene nanosheets on its surface are prone to oxidation or exfoliation, resulting in a significant decrease in absorption performance. In contrast, WPU‐MXene@FC maintained a near‐100% absorption, demonstrating that the WPU coating effectively protected the MXene during immersion. The results suggest that spraying the superhydrophobic WPU solution protects the MXene nanosheets on the fiber surface, thereby extending the chemical stability and service life of the WPU‐MXene@FC composite fabrics. Furthermore, combined with its exceptional superhydrophobic properties, WPU‐MXene@FC exhibits excellent long‐term corrosion resistance in complex service environments, laying a solid foundation for its engineering application in wearable devices, environmental protection, and electromagnetic shielding.

### 2.4 Self‐Cleaning and Wearable Stealth Application of WPU‐MXene@FC

**FIGURE 6:**
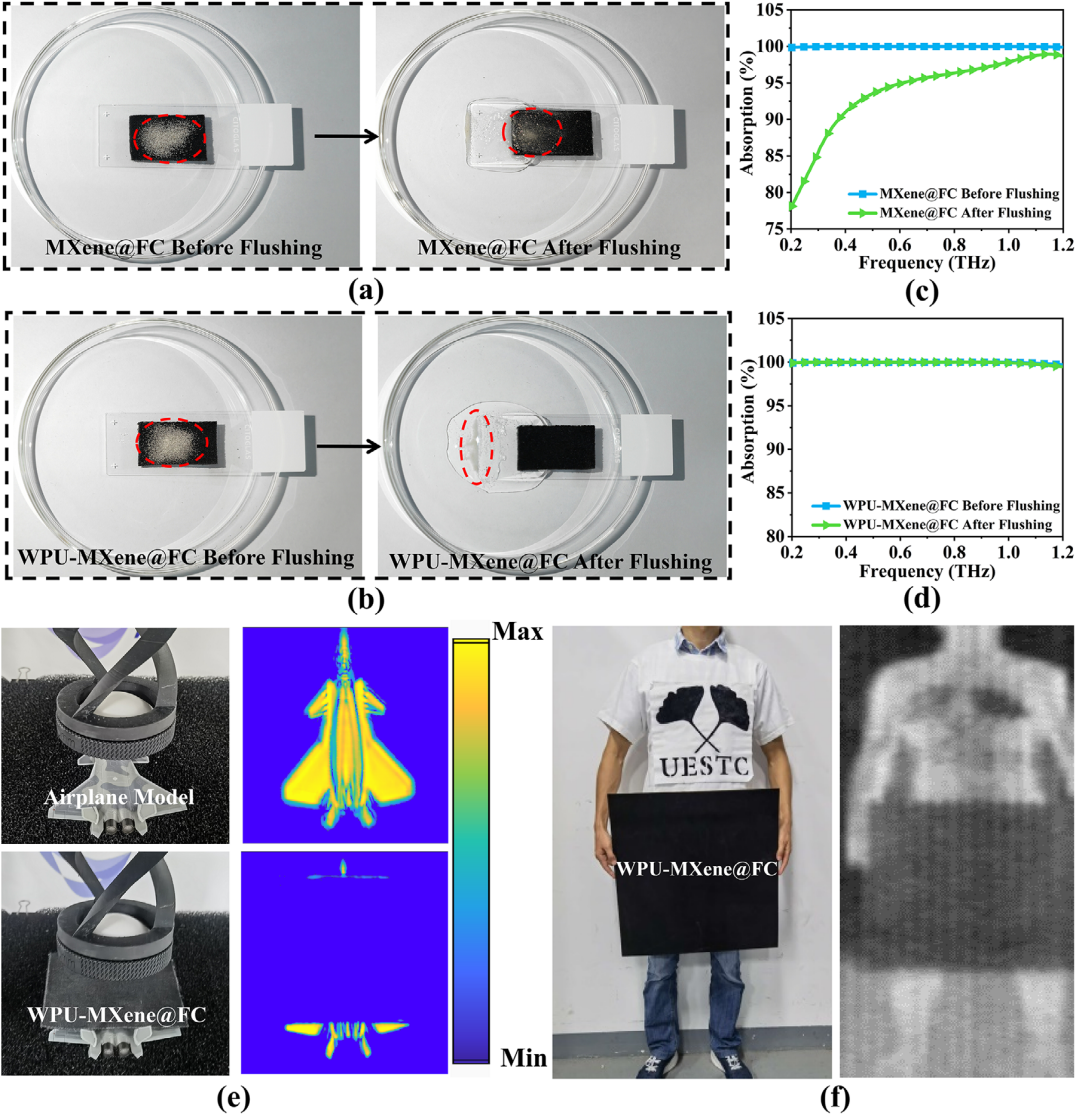
(a, b) Self‐cleaning capabilities of MXene@FC and WPU‐MXene@FC composite fabrics (the red dashed circle indicates the location of the silica powder). (c) Terahertz absorption performance of the MXene@FC before and after self‐cleaning. (d) Terahertz absorption performance of the WPU‐MXene@FC before and after self‐cleaning. (e, f) Radar imaging results of a pure aircraft model and an aircraft covered with the WPU‐MXene@FC composite fabrics.

FCs with self‐cleaning properties have a wide range of application prospects in daily life and industry [71]. Furthermore, the self‐cleaning ability of MXene@FC and WPU‐MXene@FC composite fabrics was compared and tested experimentally, as shown in Figure 6a,b. In the experiment, equal amounts of silica powder were first sprinkled on the surfaces of the two composite fabrics. The results showed that the silica powder adhered firmly to the MXene@FC surface and was difficult to fall off naturally. On the WPU‐MXene@FC surface, it showed a tendency to slide off easily. The fabrics were subsequently rinsed with an aqueous solution, and the results showed that although most of the silica powder on the MXene@FC surface was removed, some residues remained and were co‐adsorbed with the liquid on the FC surface. In contrast, the powders on the WPU‐MXene@FC surface were rapidly removed during rinsing, with the aqueous solution almost entirely flowing into the Petri dish, which fully demonstrated its excellent self‐cleaning performance. Furthermore, to verify the protective effect of WPU on terahertz absorption performance, the absorption properties of the two composite fabrics were investigated before and after cleaning (Figure 6c,d). The results revealed that the absorption performance of MXene@FC decreased significantly after rinsing. This reduction occurred because MXene nanosheets are inherently hydrophilic and, without a protective layer, tend to partially detach with the water, leading to a decrease in absorbing components on the fiber surface and inside the FC, thereby weakening its absorption performance. In contrast, the WPU‐MXene@FC protected by the WPU layer has a superhydrophobic surface, preventing aqueous solution from contacting the MXene nanosheets, resulting in no reduction in the MXene nanosheets. Consequently, the terahertz absorption performance of the WPU‐MXene@FC composite fabrics remained virtually unchanged before and after cleaning.

Furthermore, to directly verify the electromagnetic wave absorption capability of the WPU‐MXene@FC composite fabric, we conducted a stealth experiment using a radar imaging system (Figure 6e). The experimental results show that, without any covering material, the metal aircraft model exhibits a clear and complete outline in the imaging system; however, when its surface is covered with WPU‐MXene@FC, the metal outline in the covered area almost completely disappears, and the echo signal intensity is significantly reduced. This result intuitively demonstrates the composite fabric's efficient absorption and scattering suppression capability against incident electromagnetic waves. Therefore, WPU‐MXene@FC not only serves as a highly efficient electromagnetic protection material in complex environments but also shows great application potential in stealth and target weakening scenarios. It is worth further noting that this type of flexible stealth fabric is compatible with curved surfaces and irregular structures, providing new possibilities for the future development of wearable stealth equipment, flexible camouflage materials, and lightweight target detection and control technologies. Thanks to its flexibility and lightweight properties, the composite fabrics can be designed with an absorption structure with a specific pattern, achieving personalized electromagnetic stealth and protection (Figure 6f). The results revealed that the reflection signal in the “UESTC” letter region was significantly reduced, forming a clear contrast with the surrounding area. Moreover, EMWs transmission was effectively blocked, directly demonstrating its potential for applications in patterned stealth and electromagnetic shielding. Furthermore, based on the excellent bending resistance and high‐efficiency absorption performance of WPU‐MXene@FC composite fabrics, its application range can be expanded to the fields of precision detection and instrument optimization. For example, after covering the inner surface of the terahertz instrument with WPU‐MXene@FC, the noise generated by internal stray reflections and transmitted light beams can be effectively reduced, thereby significantly improving the signal‐to‐noise ratio of the system (Figure S14). In the comparison test before and after covering, the terahertz scattering power of the instrument was greatly reduced after covering, and the noise level was significantly reduced, verifying its role in suppressing interference and enhancing signal quality. This improvement mechanism is particularly important for improving the resolution of small defects in terahertz systems in precision detection [72]. Therefore, WPU‐MXene@FC not only demonstrates advantages in ultra‐wideband EMWs absorption but also has potential for application in various scenarios, including personalized stealth, precision detection, and noise suppression in optical instruments. This provides new design ideas and application prospects for the development of flexible, multifunctional electromagnetic protection fabrics.

## 3 Conclusion

In conclusion, this study developed a simple and reliable fabrication strategy for high‐performance flexible multifunctional absorption materials. Specifically, FC is activated by nitrogen plasma to introduce amine‐like nitrogen groups, which become protonated (─NH_3_^+^) under mildly acidic aqueous conditions, enabling electrostatic adsorption of negatively charged MXene. The electrostatic self‐assembly process induces a “nest‐like” structure on the fibers, which, building leverages the excellent impedance matching characteristics as well as the inherent conductivity and ohmic dissipation mechanisms of MXene nanosheets, enables high‐efficiency ultra‐broadband absorption with an EAB covering 25.3–1200 GHz. Among seven types of MXenes tested, Ti_3_C_2_ was identified as the optimal candidate due to its superior broadband absorption performance. The resulting Ti_3_C_2_‐based WPU‐MXene@FC composite fabrics achieved *RL* ≤ −30 dB in the 0.2–1.2 THz range. Notably, the composite fabrics exhibit exceptional mechanical durability and environmental stability: their *RL* remained consistently below −30 dB even after 500 bending‐folding cycles, following 48 h of immersion in acid and alkali solutions. Furthermore, experimental verification confirmed the favorable electromagnetic stealth and interference shielding capabilities of WPU‐MXene@FC. These comprehensive performances collectively underscore its broad application prospects in wearable electronics, flexible protective clothing, and military camouflage fields.

## 4 Experimental Section/Methods

### 4.1 Chemicals and Materials

Ti_3_AlC_2_ powder (99.99%, ∼400 mesh, Jilin Yiyi Technology Co., Ltd.), HF (48%–51%, Sigma–Aldrich LLC.), HCl (36%–38%, Merck), LiCl (99%, Merck), WPU (F0401, Shenzhen Jitian Chemical Co., Ltd.), FC (Taobao vendor, Hengxinyi) were used throughout the synthesis, NH_3_ (99%) was used as the plasma feed gas. Deionized (DI) water was used throughout the synthesis. All chemicals were purchased without further purification.

### 4.2 Nitrogen‐Plasma Activation of FC

The FC was cleaned with ethanol and DI water, and dried at 60°C for 12 h. Plasma treatment was performed in an RF low‐temperature plasma system (13.56 MHz). The chamber was evacuated and then filled with NH_3_ to a working pressure of 40 Pa. The FC was placed horizontally 5–8 cm away from the electrode and treated at 80 W for 10 min at 30°C. After treatment, the samples were used for subsequent adsorption within 30 min to minimize surface aging. Nitrogen plasma introduces nitrogen‐containing (amine‐like) surface functionalities, which can be protonated in mildly acidic aqueous media, rendering the fabric surface positively charged.

### 4.3 Synthesis of Various MXene Dispersions

In detail, Ti_3_C_2_ was prepared by adding 1 g of Ti_3_AlC_2_ into a mixture of 12 mL HCl, 6 mL H_2_O, and 2.5 mL HF, stirring at 35°C for 24 h, and then washing the intercalated suspension through centrifugation. To synthesize V_2_C, 1 g of V_2_AlC powder was added into 15 mL of 49% HF and stirred at 35°C for 72 h, followed by centrifugation to wash the intercalated suspension. Subsequently, 10 mL of 25% TBAOH mixed with 10 mL of H_2_O was added and intercalated at 25°C for 4 h. The intercalation solution was then washed with acetone and anhydrous ethanol to obtain multilayer V_2_C. To synthesize Mo_2_TiC_2_ and Mo_2_Ti_2_C_3_, Mo_2_TiAlC_2_ and Mo_2_Ti_2_AlC_3_ were respectively added into 15 mL of 49% HF and stirred at 40°C for 48 and 96 h, followed by centrifugation to wash the intercalated suspension. Subsequently, 10 mL of 25% TBAOH mixed with 10 mL of H_2_O was added for intercalation at 25°C for 4 h. Finally, the intercalated products were repeatedly washed with acetone to obtain multilayer Mo_2_TiC_2_ and Mo_2_Ti_2_C_3_. To synthesize Nb_4_C_3_, 1 g of Nb_4_AlC_3_ was added to 20 mL of 49% HF and stirred at 40°C for 96 h. The intercalation solution was then washed by centrifugation. The solution was then added to 10 mL of 25% TBAOH and 10 mL of H_2_O and intercalated at 25°C for 4 h. Finally, the intercalation solution was repeatedly washed with acetone to obtain multilayer Nb_4_C_3_. To synthesize Mo_2_C, Mo_2_Ga_2_C powder was added into 20 mL of 49% HF and stirred at 40°C for 96 h, followed by centrifugation to wash the intercalated suspension. Subsequently, a mixture of 10 mL of 25% TBAOH and 10 mL of H_2_O was added for intercalation at 25°C for 4 h. Finally, the intercalated product was repeatedly washed with acetone to obtain multilayer Mo_2_C. To synthesize Nb_2_C, 1 g of Nb_2_AlC powder was added to 15 mL of 40% HF and stirred at 40°C for 48 h, followed by centrifugation to wash the intercalated suspension. Subsequently, 10 mL of 25% TBAOH mixed with 10 mL of H_2_O was added for intercalation at 25°C for 4 h. Finally, the intercalated product was repeatedly washed with acetone to obtain multilayer Nb_2_C. Furthermore, to simultaneously prepare various few‐layer MXene dispersions, the multilayer MXenes obtained by freeze‐drying were first stored in a vacuum sample box. And intercalation was performed simultaneously after all multilayer MXenes had been synthesized to ensure the freshness of each dispersion.

### 4.4 Preparation of WPU‐MXene@FC

First, the prepared MXene dispersion (0–2.0 mg/mL) was placed in a magnetic stirring water bath, stirred at 600 rpm and 30°C for 1 h to prevent agglomeration. Then, the stirred MXene dispersion was poured into a Petri dish, ensuring that the liquid level was 2 mm above the thickness of the FC to maintain uniform dispersion. Subsequently, the surface‐modified FC with a thickness of approximately 1.8 mm was immersed in the MXene dispersion (at 30°C for 10 min) and repeatedly rolled with a roller (approximately 20 reciprocating motions) to press the dispersion into the FC. The FC was then removed with tweezers, placed on a dry glass plate, and freeze‐dried at −80°C for 24 h to obtain the MXene@FC composite fabric. Next, WPU emulsion was loaded into a spray bottle and uniformly sprayed onto the surface of the MXene@FC. Finally, the MXene@FC coated with WPU emulsion was placed on a low‐temperature drying glass plate and allowed to stand for 24 h to obtain the WPU‐MXene@FC composite fabric.

### 4.5 Materials Characterizations

Terahertz time‐domain spectroscopy (THz‐TDS) was measured at room temperature and 5% humidity using a commercial all‐fiber system (Fico, Zomega) with an effective spectral range of 0.2–1.2 THz at a repetition rate of 1 kHz. X‐ray diffraction (XRD) was performed using a Bruker D8 Advance XRD. Scanning electron microscopy (SEM) images were obtained using a JSM‐7600F SEM at 15 kV, and SEM energy‐dispersive spectroscopy (EDS) mapping was performed using an Ultim Max detector (Oxford Instruments) at 10 kV (Zeiss Sigma 300, Germany).

## Funding

This work is financially supported by the National Key Research and Development Program of China (No. 2023YFB3811305), the National Natural Science Foundation of China (No. 62235004, 62311530115, and 62505042), Open Foundation of China‐Belarus Belt and Road Joint Laboratory on Electromagnetic Environment Effect (No. ZBKF2024031002), the Shenzhen Science and Technology Program (No. KQTD20200820113010022), and Postdoctoral Fellowship Program of CPSF (GZC20240216).

## Conflicts of Interest

The authors declare no conflicts of interest.

## Supporting information




**Supporting File 1**: advs75438‐sup‐0001‐SuppMat.docx.

**Supporting File 2**: advs75438‐sup‐0002‐VideoS1.mp4.

## Data Availability

The data that support the findings of this study are available from the corresponding author upon reasonable request.
