# Supplementary material for: Electrostatic Self‐Assembly Induced Nest‐Like MXene Networks on Fabric for Ultra‐Broadband Flexible Absorption
Source: Adv Sci (Weinh). 2026 May 8;13(40):e75438. doi: 10.1002/advs.75438 (PMC13335453; doi:10.1002/advs.75438)
Supplement: Supplementary file 1 — Supporting File 1: advs75438‐sup‐0001‐SuppMat.docx. [file ADVS-13-e75438-s002.docx]

***Supporting Information***

**Electrostatic Self-Assembly Induced Nest-like MXene Networks on Fabric for Ultra-Broadband Flexible Absorption**

**Min Luo^a, b^, Zihao Chen^a^, Haotian Li^a^, Kexun Li^c^, Wei Wang^c^, Weishi Jiao^a^, Donghong Wang^c^, Qiye Wen^a, b, d,*^**

*^a^ School of Electronic Science and Engineering, University of Electronic Science and Technology of China, Chengdu 610054,* *China*

*^b^ Tianfu Jiangxi Laboratory, Chengdu 641419, China*

*^c^ China-Blarus Belt and Road Joint Laboratory on Electromagnetic Environment Effect, The 33rd Research Institute of China Electronics Technology Group Corporation, Taiyuan, 030032, PR China*

*^d^ Shenzhen Institute for Advanced Study, University of Electronic Science and Technology of China, Shenzhen 518110, China*

**Corresponding Email: qywen@uestc.edu.cn*

**The content of supplementary material:**

Total number of figures: 14

Total number of tables: 2

Terahertz time-domain spectrum (THz-TDS) was measured by a commercial all-fiber system (Fico TM, Zomega), with effective spectral range 0.2~1.2 THz and a repetition rate of 1 kHz. Through the fast Fourier transform of time domain signal, the amplitude and phase of the wave can be obtained, so as to accurately extract the *RL* of the material in terahertz region. The *RL* of the sample in this area can be calculated according to the following formula:

| $\text{ }\text{RL}\text{ }\left( \text{dB} \right)\text{=}\text{-}\text{20}\text{log}_{\text{10}}\text{(\vert}\text{E}_{\text{i}}\text{\vert/\vert}\text{E}_{\text{r}}\text{\vert)}$ (1) |
| --- |

Where $\text{E}_{\text{i}}$ and $\text{E}_{\text{r}}$ are the amplitudes of the reflected terahertz pulse of the test sample and the reference aluminum plate, respectively.

To quantitatively assess the material's coupling capability to EMWs and its intrinsic dissipation capability, we calculated the normalized intrinsic impedance and attenuation constant based on the measured complex permittivity. For fabric-based composite systems without magnetic fillers, the relative permeability can be reasonably approximated as μ_r_ ≈ 1 in the terahertz band. The normalized intrinsic impedance is used to measure the ease with which incident electromagnetic waves couple from free space into the material, and is defined as:

| $\text{ }\left\vert\frac{Z}{Z_{0}} \right\vert\text{=}\left\vert\sqrt{\frac{\mu_{r}}{\varepsilon_{r}}} \right\vert\approx\left\vert\frac{1}{\sqrt{\varepsilon_{r}}} \right\vert$ (2) |
| --- |

Where Z is the intrinsic impedance of the material, Z_0_$\approx$377 Ω is the free-space impedance, and $\varepsilon_{r}$ is the complex permittivity. When $\left| \frac{Z}{Z_{0}} \right|$ approaches 1, the impedance mismatch at the air-material interface weakens, interface reflection decreases, and more electromagnetic waves can enter the absorber, thus providing the energy basis for subsequent losses. The attenuation constant α reflects the energy attenuation capability of EMWs propagating inside the material and can be calculated by the following formula:

| $\text{ }\text{α}\text{=}\frac{2\pi f}{c}\sqrt{\frac{\mu_{r}\varepsilon^{'}}{2}(\sqrt{1+{(\frac{\varepsilon^{''}}{\varepsilon^{'}})}^{2}})-1})$ (3) |
| --- |

Where *f* is the frequency and c is the speed of light in a vacuum. A larger α indicates that the energy decays faster per unit propagation distance, which helps to suppress the transmission of EMWs.

To evaluate the broadband electromagnetic absorption performance of absorbing materials, we introduce the key parameters: average reflection loss (*RL_ave_*). There are calculated using the equations as follows:

| ${\text{ }\text{RL}}_{\text{ave}}\text{ }\text{(}\text{dB}\text{)=}\frac{\text{1}}{\text{N}}\sum_{\text{i}\text{=1}}^{\text{N}} \text{RL}\text{(}\text{f}_{\text{i}}\text{)}$ (4) |
| --- |

where *N* is the number of frequency points, $\text{RL}\text{(}\text{f}_{\text{i}}\text{)}$ represents the *RL* value at the ith frequency point. A higher *ARL* value indicates strong absorption across the entire measurement range.

**Supplementary Figures**


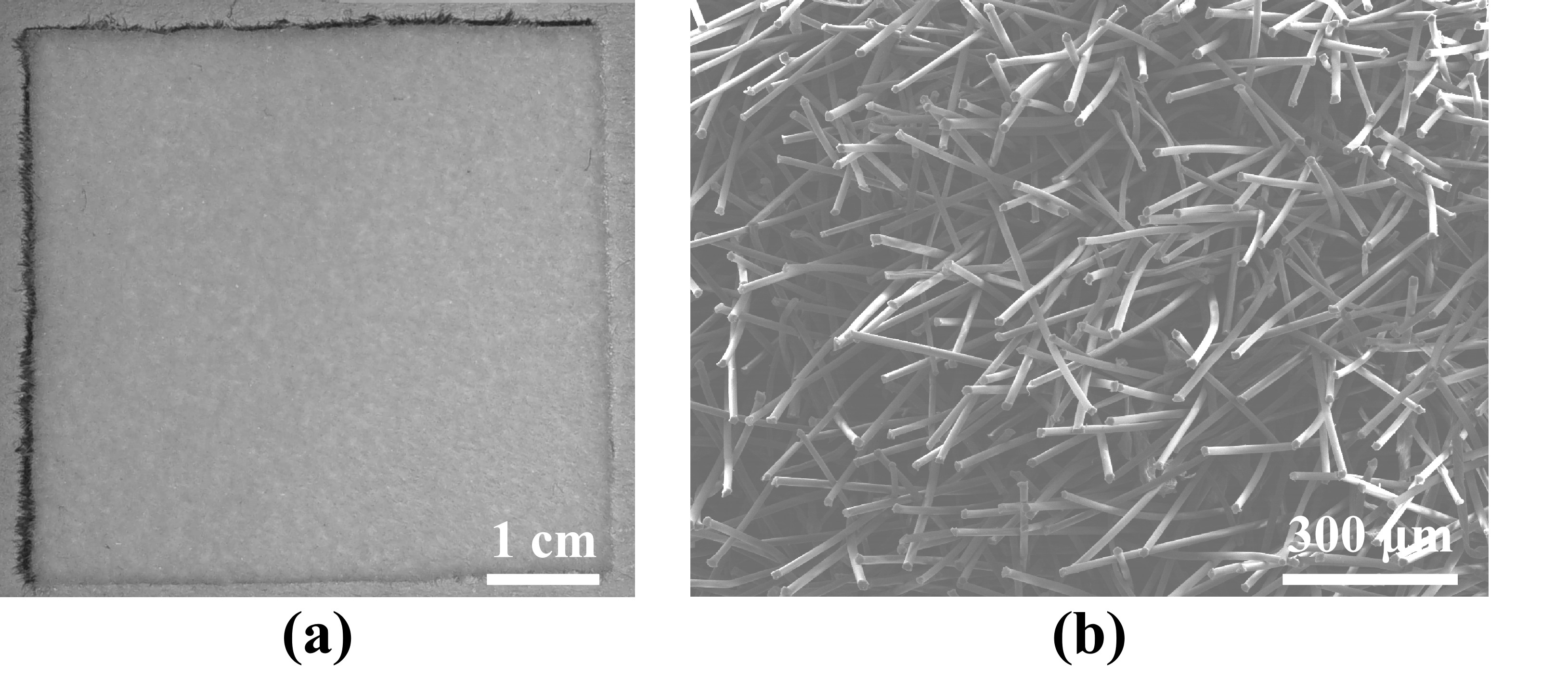


**Figure S1.** (a) Optical pictures of the fabric. (b) SEM characterization of fabric.


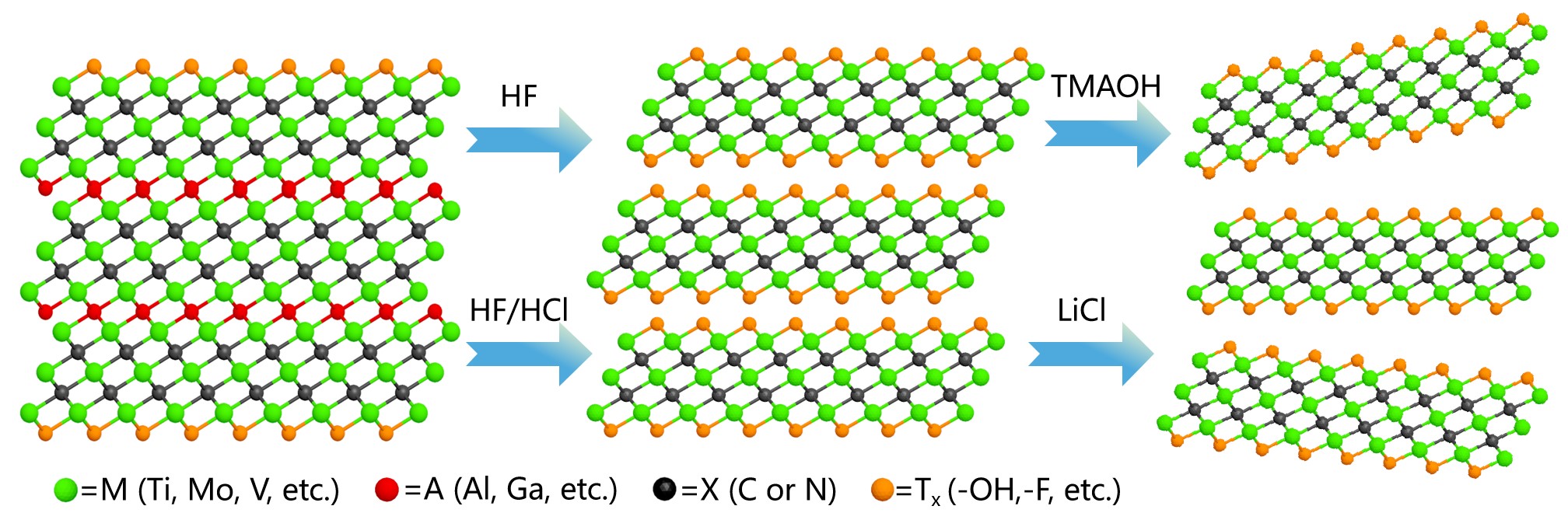


**Figure S2.** Schematic diagram of preparation process of MXene.


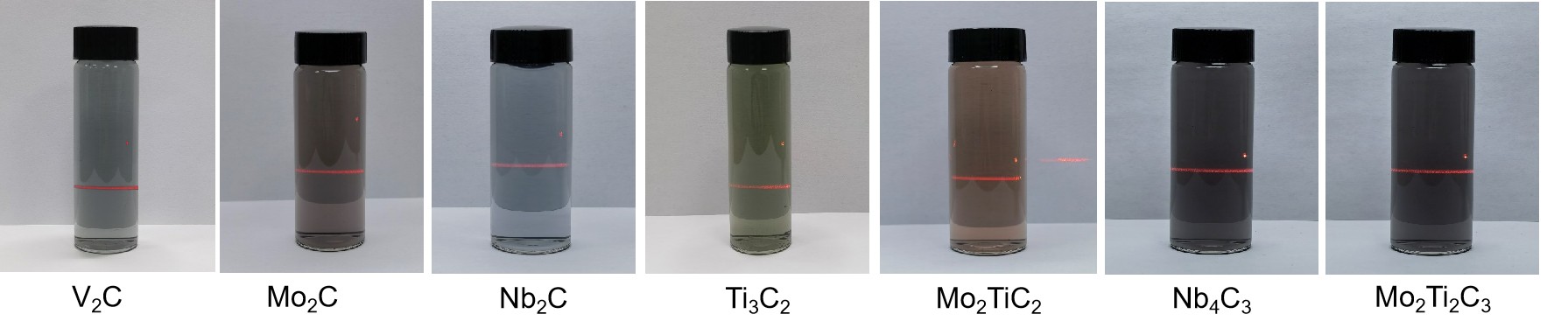


**Figure S3.** Display of seven different MXene dispersions.


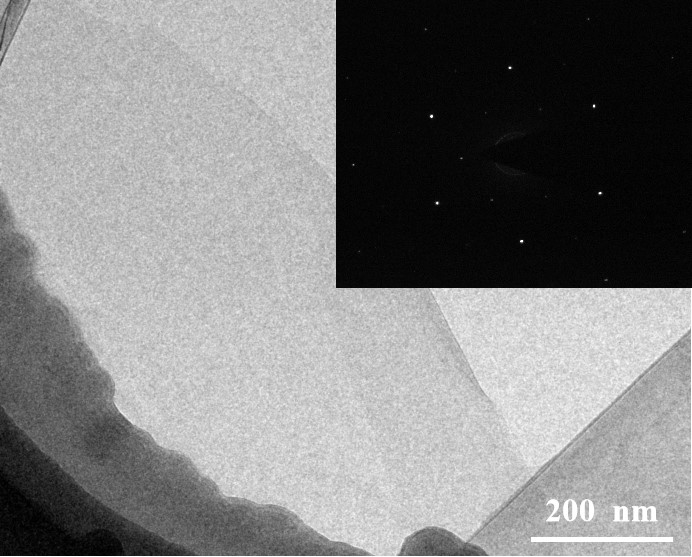


**Figure S4.** Transmission electron microscopy (TEM) image of a single-layer MXene; the upper right corner shows selected area electron diffraction (SAED).


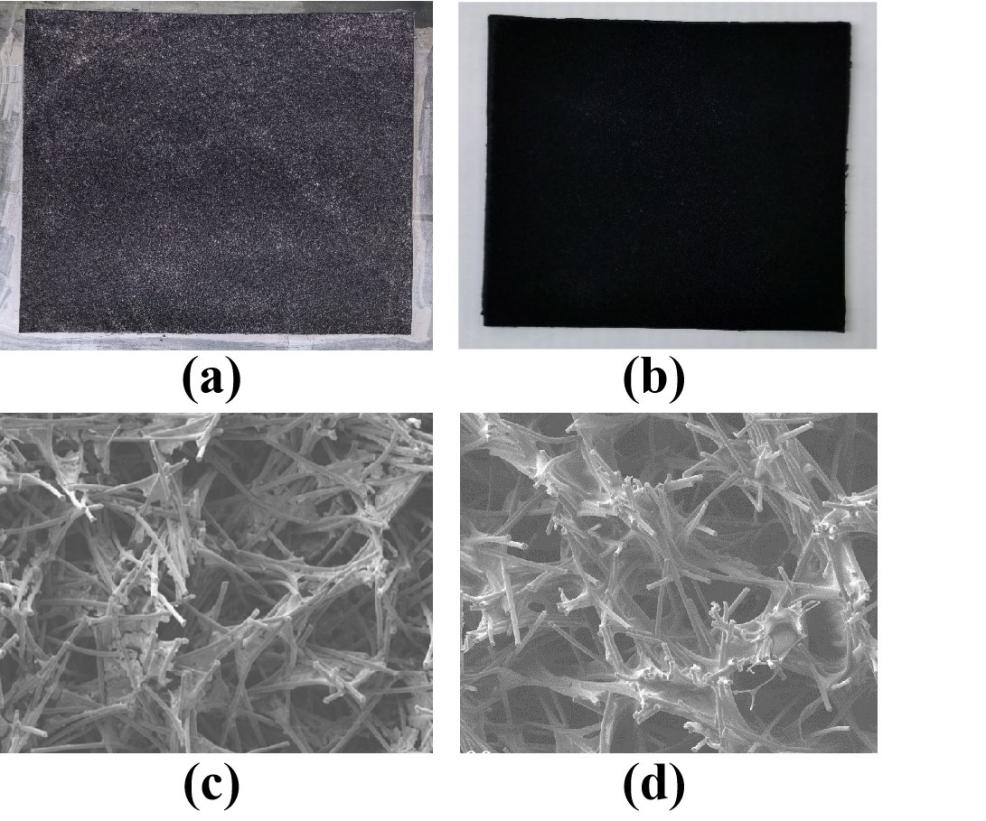


**Figure S5.** (a) MXene@FC optical image. (b) WPU-MXene@FC optical image.


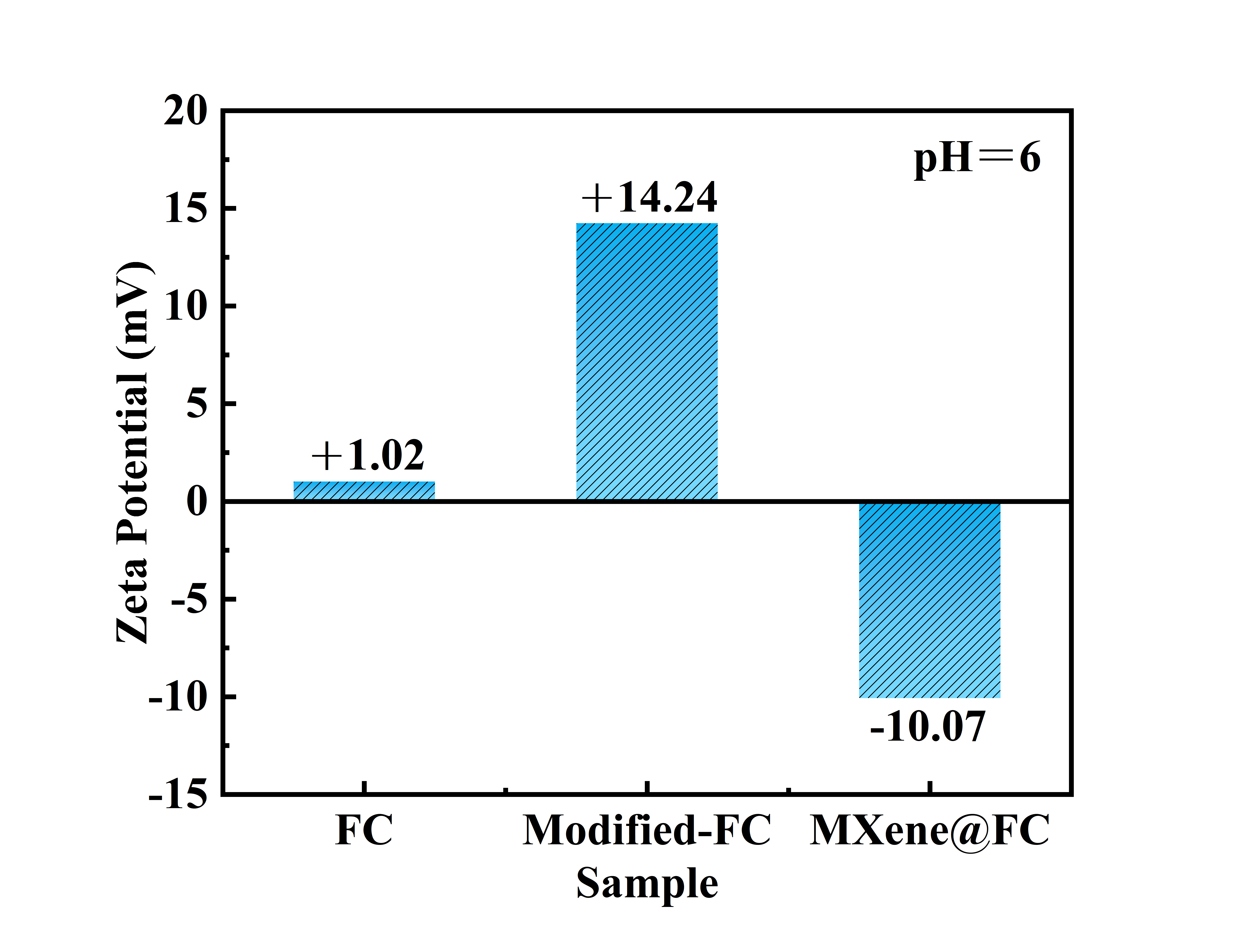


**Figure S6.** Zeta potential of FC, Modified-FC and MXene@FC at pH=6.


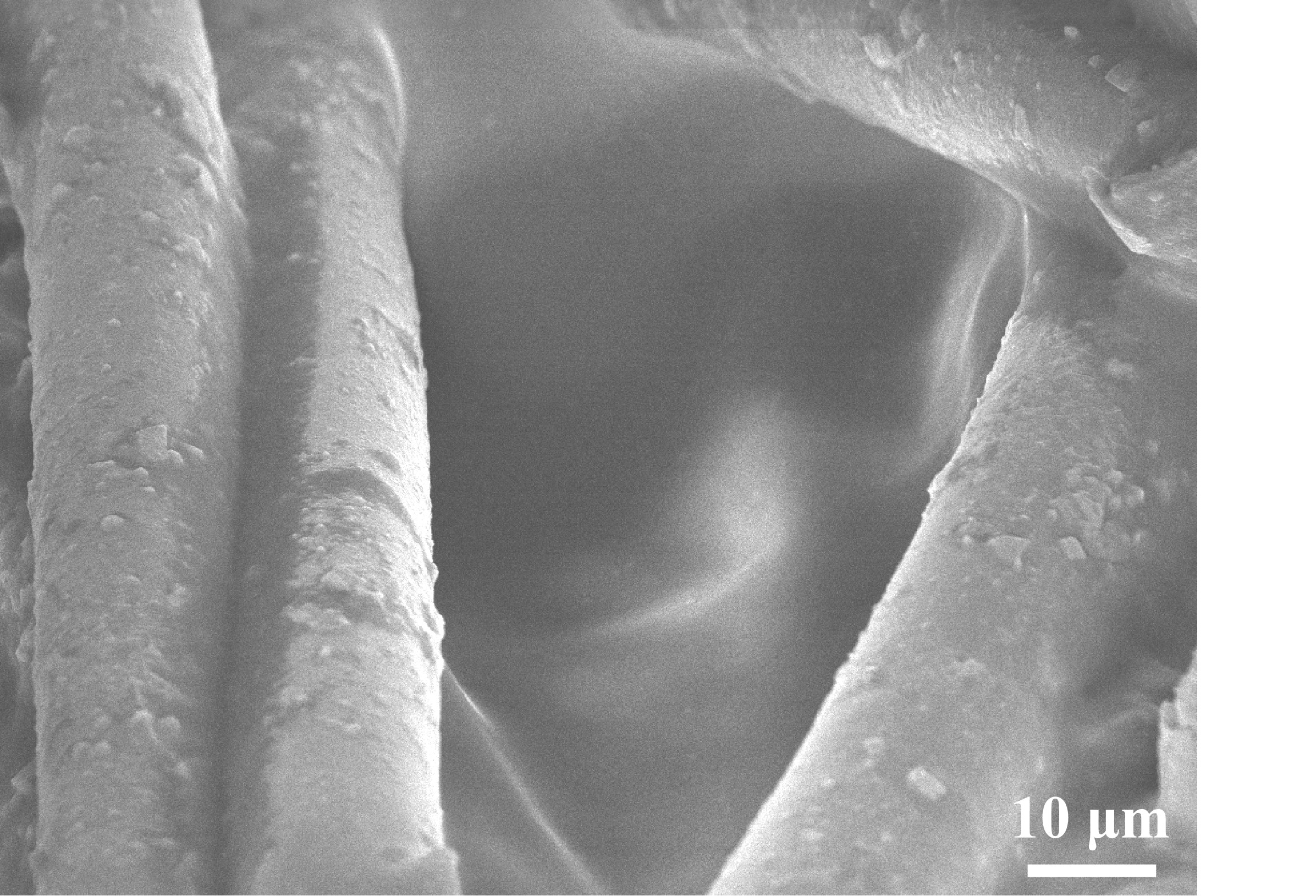


**Figure S7.** High-magnification SEM image of WPU-MXene@FC.


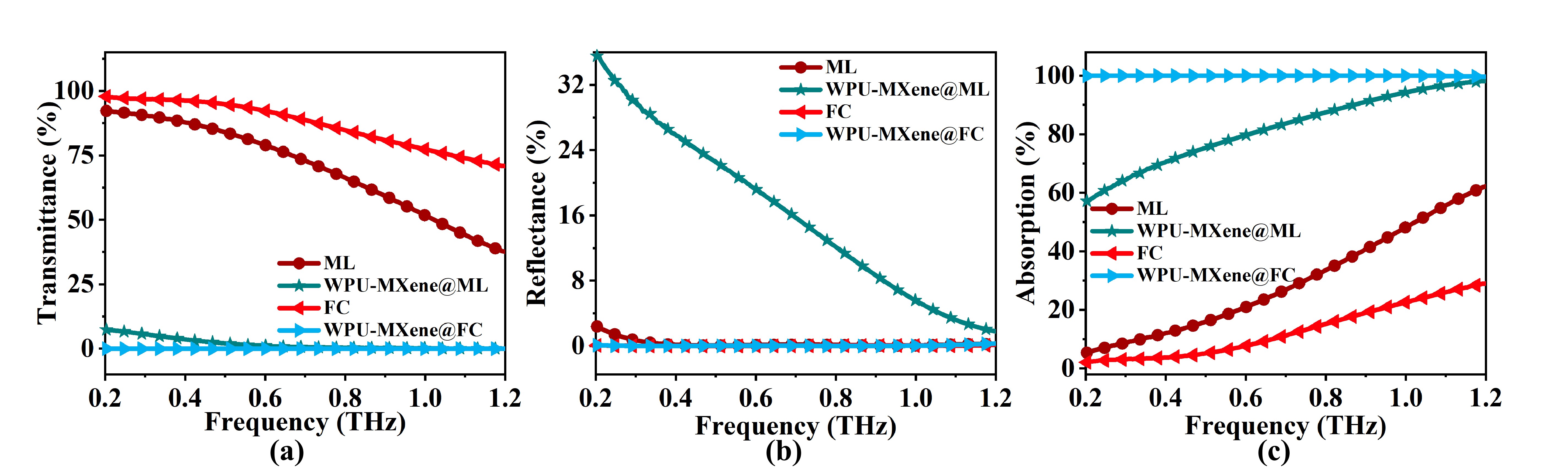


**Figure S8.** Transmission efficiency (a), reflection efficiency (b), and absorption efficiency (c) of fabrics made of different materials when incident with EMWs in the 0.2–1.2 THz range.


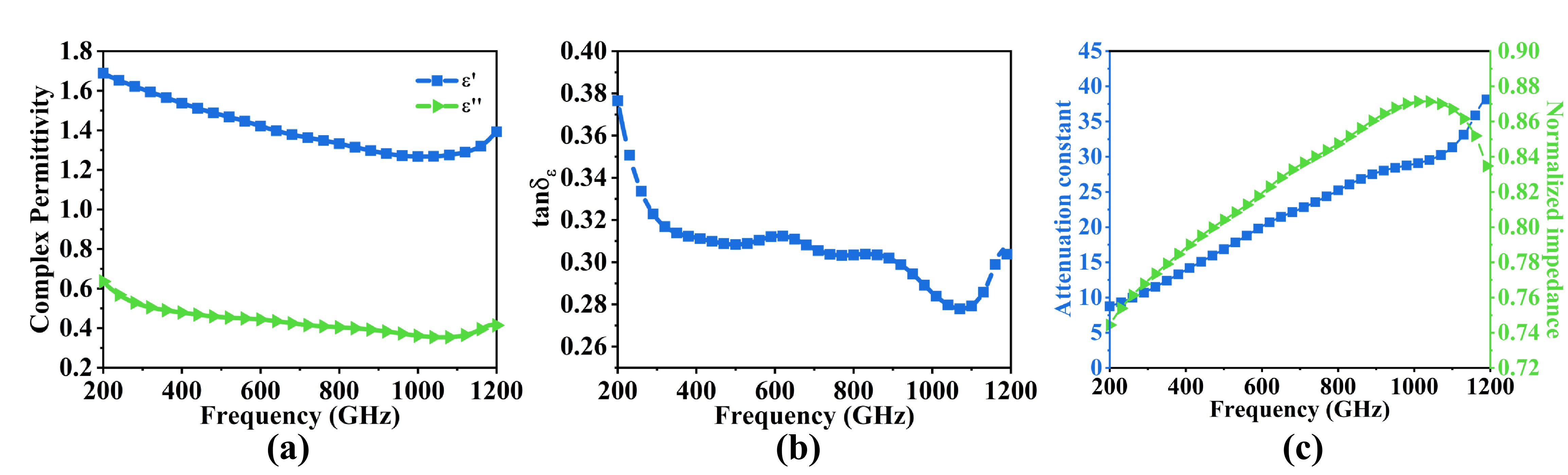


**Figure S9.** (a) Complex dielectric constant (εʹ and ε"), (b) dielectric loss tangent (tan δ), (c) attenuation constant and normalized impedance (|Z/Z_0_|) of WPU-MXene@FC composite fabric in the 200–1200 GHz range.


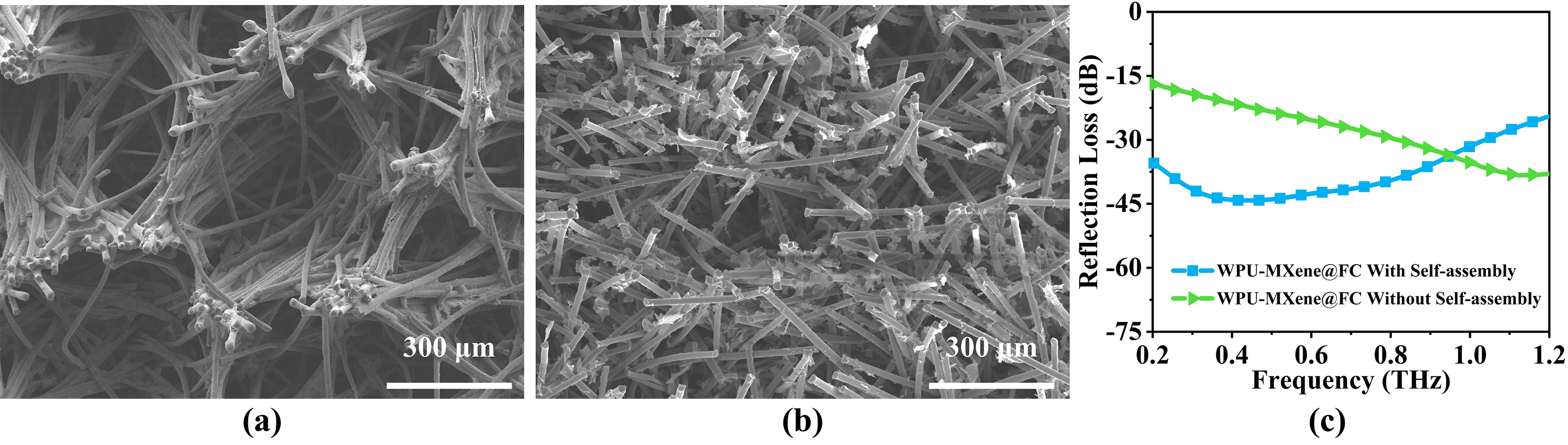


**Figure S10.** SEM images of the two samples were obtained using (a) electrostatic self-assembly and (b) without electrostatic self-assembly. (c) The corresponding RL curves of WPU-MXene@FC in the 0.2–1.2 THz range.


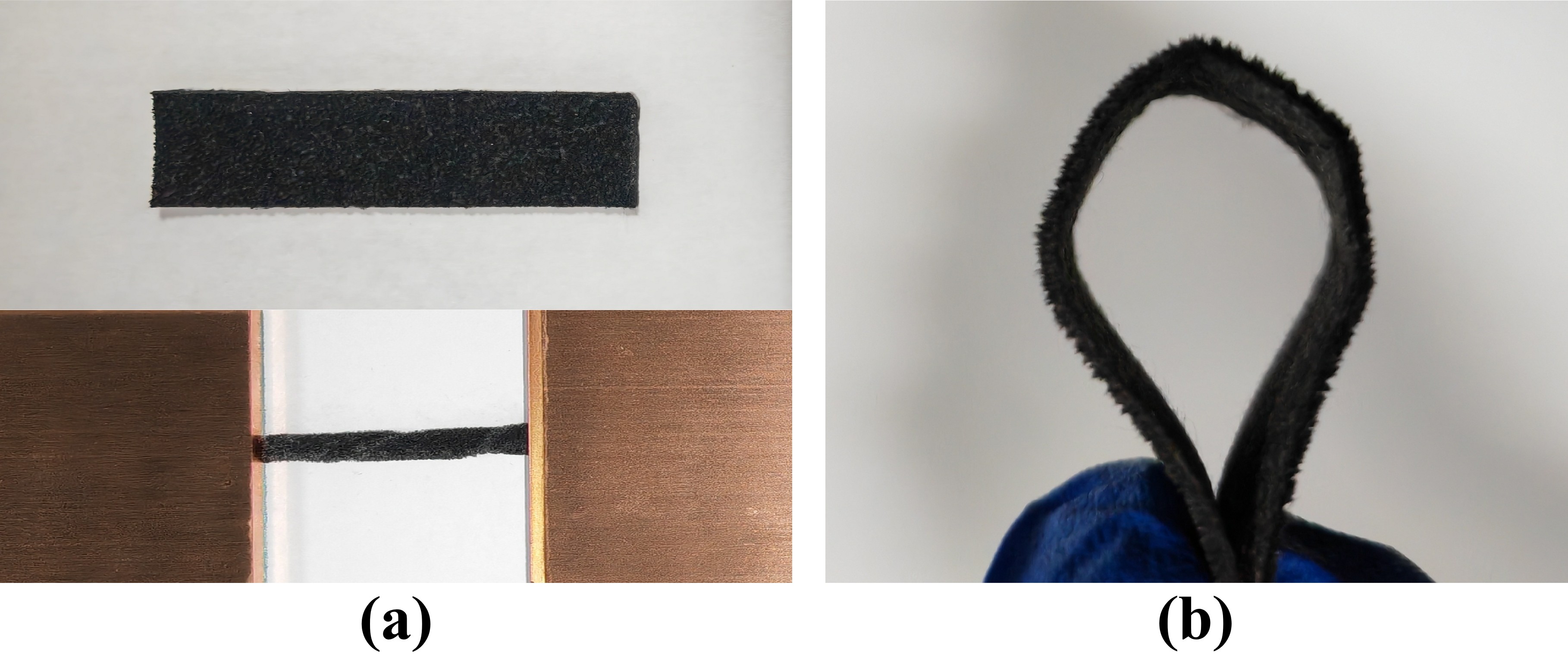


**Figure S11.** Schematic diagram of the anti-torsion properties (a) and bending resistance (b) of WPU-MXene@FC composite fabric.


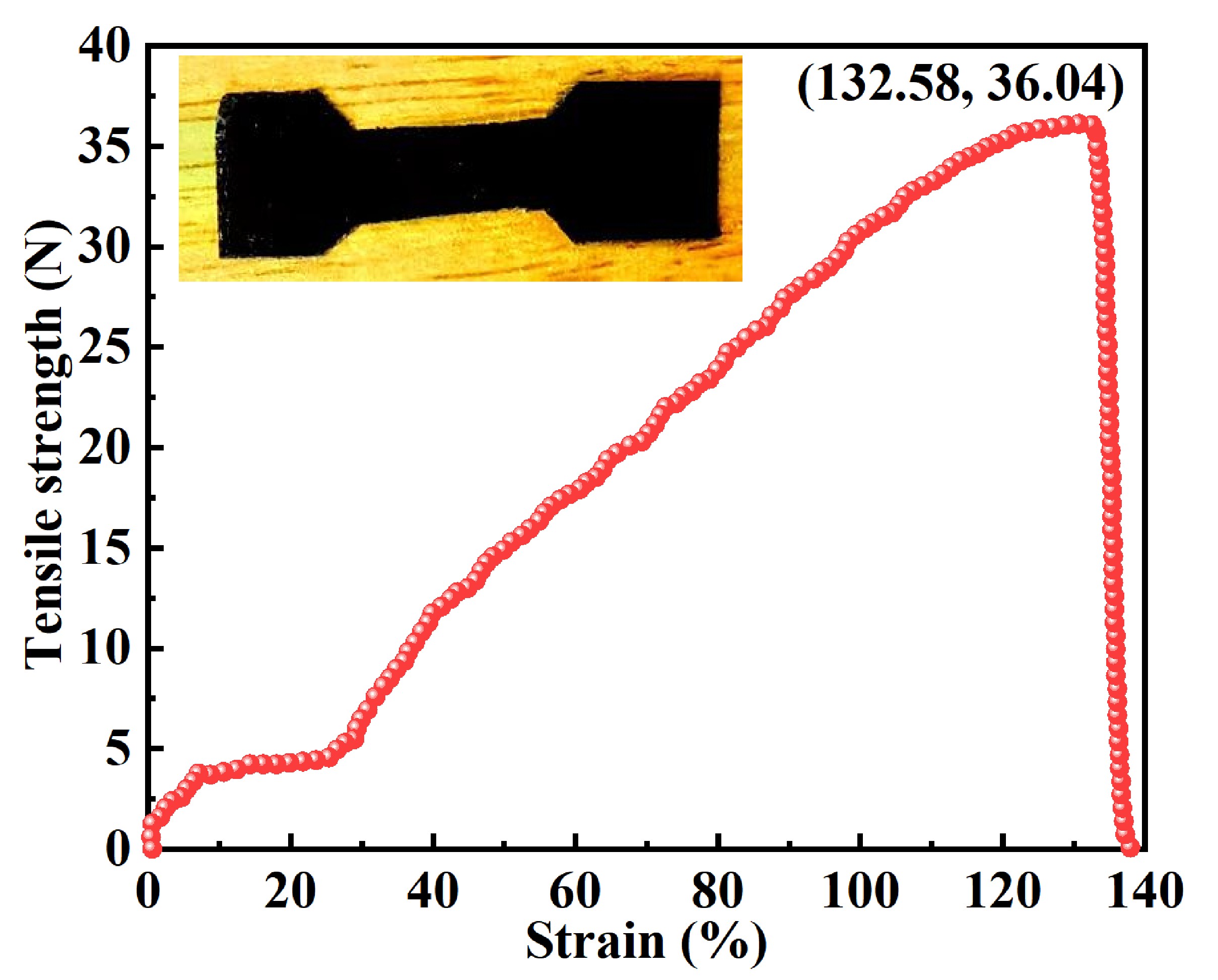


**Figure S12.** Tensile strain curve of WPU-MXene@FC composite fabric.

**
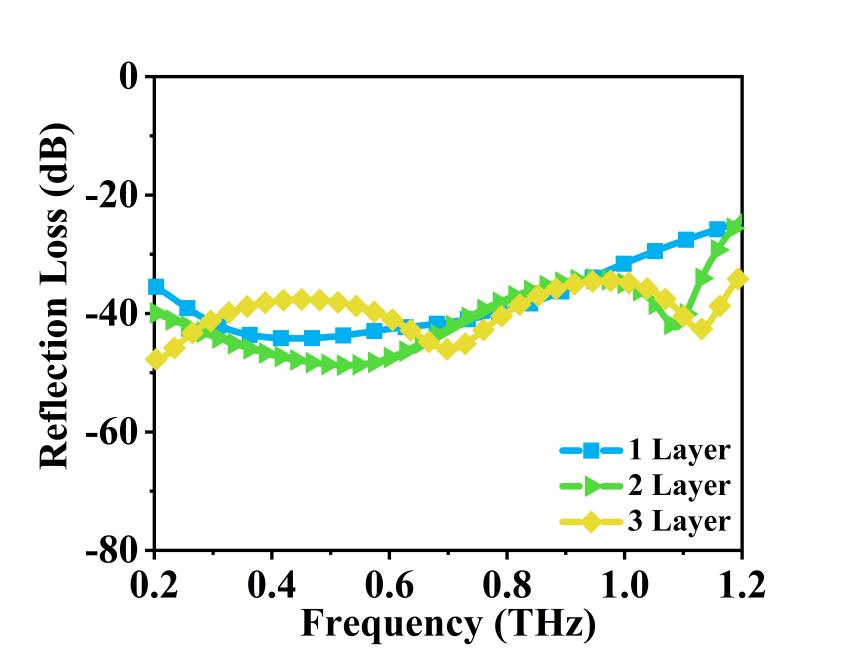
**

**Figure S13.** Effect of stacking layer number (1–3 layers) on the RL of WPU-MXene@FC in the 0.2–1.2 THz range.


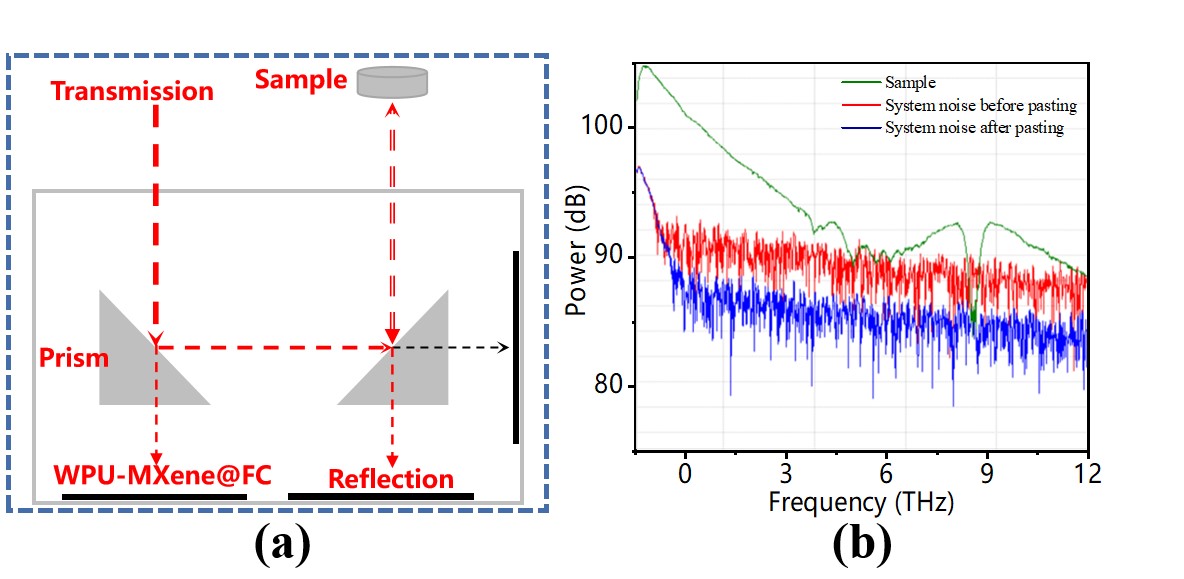


**Figure S14.** (a) A scheme for improving the signal-to-noise ratio of a terahertz instrument. The black solid line represents the WPU-MXene@FC composite fabric. (b) Terahertz scattering power before and after covering the instrument's inner surface with the WPU-MXene@FC composite fabric.

**Table S1.** Seven MXene synthesis processes

| MAX | MXene | Etching Agent | Interlayer Liquid | Detergent |
| --- | --- | --- | --- | --- |
| Ti_3_AlC_2_ | Ti_3_C_2_ | HCl/HF | LiCI/H_2_O | DI water |
| V_2_AlC | V_2_C | HF | TBAOH/H_2_O | Acetone / EtOH |
| Nb_2_AlC | Nb_2_C | HF | TBAOH/H_2_O | Acetone |
| Mo_2_Ga_2_C | Mo_2_C | HF | TBAOH/H_2_O | Acetone |
| Mo_2_TiAlC_2_ | Mo_2_TiC_2_ | HF | TBAOH/H_2_O | Acetone |
| Nb_4_AlC_3_ | Nb_4_C_3_ | HF | TBAOH/H_2_O | Acetone |
| Mo_2_Ti_2_AlC_3_ | Mo_2_Ti_2_C_3_ | HF | TBAOH/H_2_O | Acetone |

**Table S2.** Comparative example of WPU-MXene@FC composite fabric and other absorbing materials

| **Materials** | **Thickness (mm)** | **EAB (GHz)** | **RL_min_ (dB)** | **Flexible** | **Ref** |
| --- | --- | --- | --- | --- | --- |
| Ag-Cu alloy-based | 20 | 37  (3~40 GHz) | -24.5 | × | [1] |
| SiC ceramic | 18 | 34.64  (4.85~39.49 GHz) | -45.9 | × | [2] |
| CIP/MWCNT | 2.9 | 35  (75~110 GHz) | -37.04 | √ | [3] |
| 3D Graphene Foam | 4 | 1045  (0.155-1.2 THz) | -19 | × | [4] |
| graphene/MWCNT | 8 | 25.7  (5.8~18 GHz/ 26.5~40 GHz) | -35.68 | × | [5] |
| Fe/C | 2.5 | 4.4 | -47.1 | √ | [6] |
| SiC@FC | 2.8 | 8.78  (9.20–17.98 GHz) | -48.4 | √ | [7] |
| Water-based resonator | 5.1 | 83.48(16.52~100.00 GHz) | -23.5 | × | [8] |
| WPU-MXene@FC | 1.8 | 1174.7  (25.3~1200 GHz) | -44.2 | √ | **This Work** |

1. Yang Z, Liang Q, Duan Y, et al. Electromagnetic characteristics and 3D-printing realization of a lightweight hierarchical wave-absorbing metastructure for low-frequency broadband absorption[J]. Journal of Alloys and Compounds, 2023, 949: 169894.
2. Wang W, Li Z, Gao X, et al. Material extrusion 3D printing of large-scale SiC honeycomb metastructure for ultra-broadband and high temperature electromagnetic wave absorption[J]. Additive Manufacturing, 2024, 85: 104158.
3. Wang Y, Su R, Chen J, et al. 3D printed bioinspired flexible absorber: toward high-performance electromagnetic absorption at 75–110 GHz[J]. ACS applied materials & interfaces, 2023, 15(46): 53996-54005.
4. Huang Z, Chen H, Huang Y, et al. Ultra‐broadband wide‐angle terahertz absorption properties of 3D graphene foam[J]. Advanced functional materials, 2018, 28(2): 1704363.
5. Zhang Q, Du Z, Hou M, et al. Ultralight, anisotropic, and self-supported graphene/MWCNT aerogel with high-performance microwave absorption[J]. Carbon, 2022, 188: 442-452.
6. Cheng J B, Zhao H B, Zhang A N, et al. Porous carbon/Fe composites from waste fabric for high-efficiency electromagnetic wave absorption[J]. Journal of Materials Science & Technology, 2022, 126: 266-274.
7. Xing Z, You X, Ouyang H, et al. Porous and lightweight continuous SiC fiber reinforced Si3N4–SiC composites for wide frequency electromagnetic wave absorption[J]. Composites Part B: Engineering, 2025, 300: 112497.
8. Deng G, Chen W, Yu Z, et al. 3D-printed dielectric-resonator-based ultra-broadband microwave absorber using water substrate[J]. Journal of Electronic Materials, 2022, 51(5): 2221-2227.
